# Vegetable Additives in Food Packaging Polymeric Materials

**DOI:** 10.3390/polym12010028

**Published:** 2019-12-22

**Authors:** Silvestru Bogdănel Munteanu, Cornelia Vasile

**Affiliations:** 1 Faculty of Physics, Alexandru Ioan Cuza University, 11 Carol I bvd, 700506 Iasi, Romania; 2 “P. Poni” Institute of Macromolecular Chemistry, Romanian Academy, 41A Grigore GhicaVoda Alley, 700487 Iasi, Romania; cvasile@icmpp.ro

**Keywords:** lignocellulosic fibers, nano-cellulose, lignin, plant extracts, polysaccharides, chitosan, starch, antioxidant, antimicrobial, additive, reinforcement

## Abstract

Plants are the most abundant bioresources, providing valuable materials that can be used as additives in polymeric materials, such as lignocellulosic fibers, nano-cellulose, or lignin, as well as plant extracts containing bioactive phenolic and flavonoid compounds used in the healthcare, pharmaceutical, cosmetic, and nutraceutical industries. The incorporation of additives into polymeric materials improves their properties to make them suitable for multiple applications. Efforts are made to incorporate into the raw polymers various natural biobased and biodegradable additives with a low environmental fingerprint, such as by-products, biomass, plant extracts, etc. In this review we will illustrate in the first part recent examples of lignocellulosic materials, lignin, and nano-cellulose as reinforcements or fillers in various polymer matrices and in the second part various applications of plant extracts as active ingredients in food packaging materials based on polysaccharide matrices (chitosan/starch/alginate).

## 1. Introduction

Pristine polymeric materials alone often show poor physico-chemical properties. The incorporation of additives into polymeric materials improves their processability, tuning their properties to make them suitable for multiple applications like packaging, automotive, design, constructions, etc. Additivation of various compounds or nanoparticles to the virgin polymers can improve both bulk and surface properties of the products. According to the European Community an additive is “a substance which is incorporated into plastics to achieve a technical effect in the finished product, and it is intended to be an essential part of the finished article” [1].

Efforts are made to incorporate into the raw polymers various biodegradable additives with a low environmental fingerprint, such as by-products and biomass. Therefore, biobased, biodegradable polymer composites are more and more studied, as a large number of biodegradable polymers are already commercially available [2].

Composite materials exhibit advantages from the combination of multiple properties, which cannot be achieved by a monolithic material as they are systems which consist of one or more discontinuous phases enclosed in a continuous matrix [3]. The discontinuous, disperse phase, which is completely immiscible with the matrix, can be a reinforcement (reinforcing agent) or filler and the resultant composite shows optimized mechanical properties, such as strength, stiffness, and hardness [4]. As traditional plastics are resistant to biodegradation, the concept of using natural plastics (natural biodegradable polymers or biopolymers) as reinforced matrices for biocomposites is getting more and more interest.

In this review various recent applications of plant-based additives (lignocellulosic fibers/nano-cellulose as well as bioactive plant extracts) as reinforcements and active ingredients in food packaging materials are illustrated. Natural polysaccharide biopolymers (such as chitosan/starch/alginate) are nontoxic, biodegradable, biocompatible, and largely used in food packaging: Chitosan is known for its broad antimicrobial activity and its excellent film-forming properties; alginates have good film-forming properties, retain moisture, reduce microbial counts, and retard oxidative off-flavors; and starch is also particularly important for its cheap price and its frequency in nature. For these reasons, this review will present applications of plant extracts as active ingredients in natural polysaccharide biopolymers: chitosan/starch/alginate.

### Classification of Natural Biodegradable Polymers and Additives

Natural biodegradable polymers and additives are polymers formed naturally by the living organisms by enzyme-catalyzed reactions and reactions of chain growth from monomers which are formed inside the cells by complex metabolic processes [5].

Natural additives can be high molecular weight (natural polymers), such as proteins (collagen, silk, and keratin), carbohydrates (starch and glycogen), lignin, cellulose, high molecular weight phenolics (tannins and derivatives), and low molecular weight active substances, such as cold-pressed oils, essential oils (organic volatile compounds, generally of low molecular weight, containing phenols, alcohols, ethers or oxides, aldehydes, ketones, esters, amines, amides, heterocycles, and terpenes [6]), or low molecular weight phenolics (phenolic acids and flavonoids) [7]. Natural additives are widely used materials in many applications in combination with synthetic or natural polymers. These materials, together with pomaces and biowaste, are nontoxic, less expensive than the synthetic ones, ecologically friendly, and widely available.

**Figure 1:**
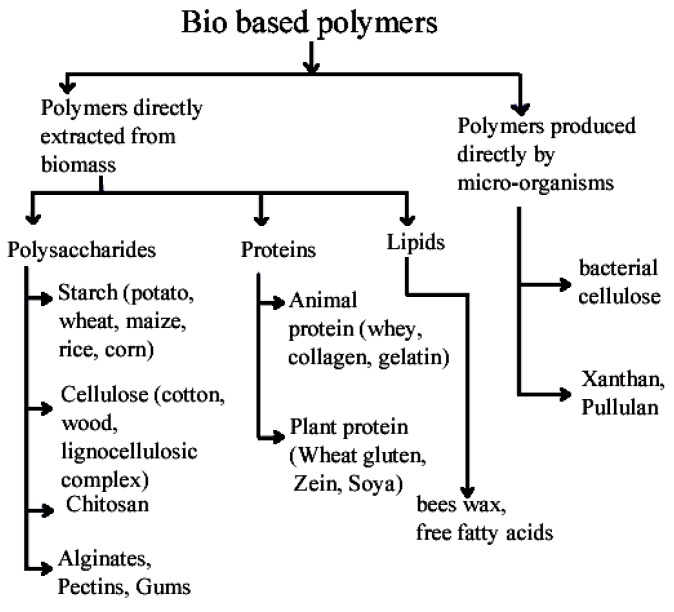
Classification of bio-additives (adapted from [8]).

**Figure 2:**
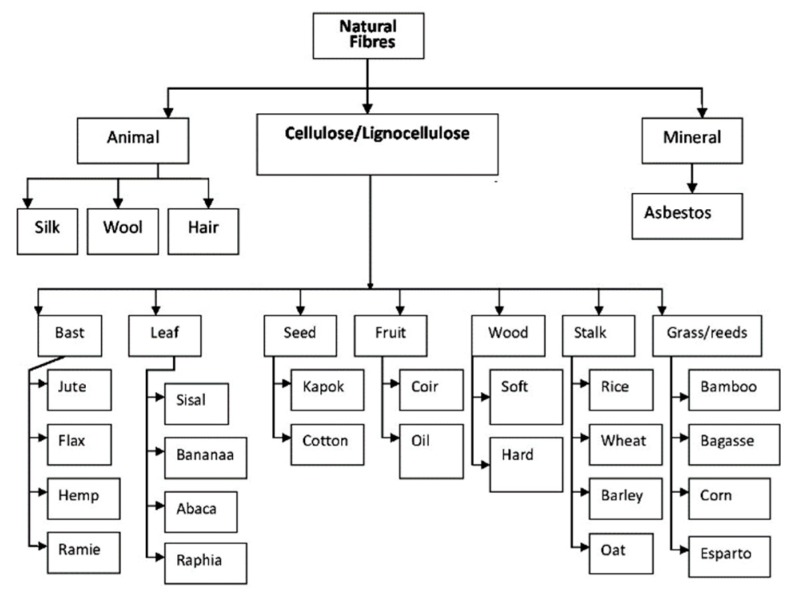
Classification of natural fibers (adapted from [9]).

The natural polymeric additives and fibers can be classified according their origin into polymers extracted from biomass or produced by micro-organisms (Figure 1 [8]) or obtained from vegetable (plant), animal, or mineral sources (Figure 2; [9]). Biopolymers that are biobased and bio-degradable include polylactic acid (PLA), polyhydroxyalkanoates (PHA: Poly(3-hydroxybutyrate) (PHB), poly(3-hydroxybutyrate-*co*-3-hydroxyvalerate) (PHBV), and poly(3-hydroxybutyrate-*co*-4-hydroxybutyrate) (PHBHB)) derived mainly from microorganisms and thermoplastic starch (TPS)-based materials. Aliphatic polyesters are also used because of their biodegradability and include poly(glycolic acid) (PGA)) and poly(alkylene dicarboxylate)s (such as poly(butylene succinate) (PBS) and poly(butylene adipate-co-terephthalate) (PBAT)), derived from both fossil fuel and renewable resources [8].

Mineral and animal fibers such as hair silk and fibers are not widely used as reinforcements - but plant fibers have been used widely in biocomposites field for applications in the areas of automotive, marine and construction [10].

The most interesting fibers for composite reinforcements and most commonly accepted fibers by the industry [11] are from plants, in particular bast, leaf, and wood fibers. The fibers are basically a rigid, crystalline cellulose microfibril-reinforced amorphous lignin and/or hemicelluloses matrix. As the environment is concerned, these fibers are biodegradable, light weight, relatively cheap, and are “carbon positive” since they absorb more carbon dioxide than they release. In thermoplastics they are introduced by melt mixing and in thermosets by vacuum-assisted transfer molding (VARTM) and vacuum bag resin transfer molding (RTM) [12].

In Table 1, recent data regarding the applications of the most-used natural polymers and natural extracts used as components/additives in new performant materials, based on their source and type (animal/vegetable/mineral and low/high molecular weight), are summarized, together with the corresponding obtaining and mixing methods. As can be seen, the natural additives are widely used as filler and additive materials to improve the biodegradability or the mechanical properties (reinforcements) or to provide antioxidant or antibacterial activity in various synthetic or natural polymeric matrices. These natural additives are added to the polymeric matrices mainly by melt blending and solution casting.

## 2. Lignocellulosic Materials and Plant Extracts in Polymeric Composites (As Reinforcements/Components/Additives)

In this review, various applications of plants used as bioresources are discussed, providing valuable raw materials used as additives in polymeric matrices, such as lignocellulosic fibers [69] and nano-cellulose [70], as well as plant extracts containing bioactive phenolic compounds [42] used in the pharmaceutical and cosmetic industries [71]. The increasing environmental concerns draw attention on their use as active compounds, instead of synthetic ones.

In this review we will illustrate in the first part (Section 2.1) recent examples of lignocellulosic materials, lignin, and nano-cellulose as reinforcements or fillers in various polymer matrices, and in the second part (Section 2.2) various applications of plant extracts as active ingredients in food packaging materials based on polysaccharide matrices (chitosan/starch/alginate).

### 2.1. Lignocellulosic Materials, Lignin and Nano-Cellulose As Reinforcements (Additives) in Polymer Matrices

**Figure 3:**
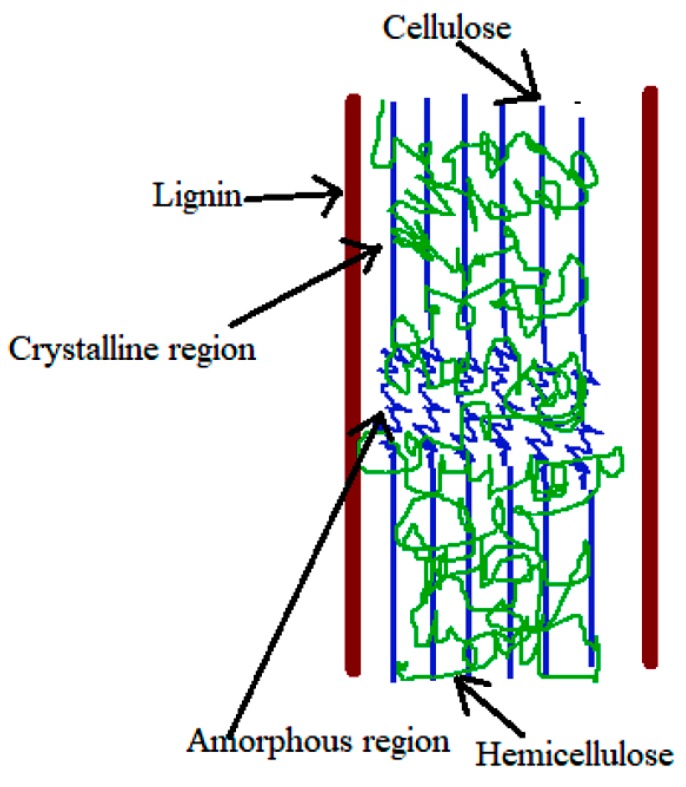
Structure of lignocellulosic biomass ([73]).

Lignocellulosic biomass, mainly composed of cellulose, hemicellulose, and lignin (Figure 3), is the most abundant plant material and therefore is the most promising feedstock being renewable, inexpensive, biodegradable and non-toxic. Due to the environmental concerns, the development of composite polymeric materials containing lignocellulosic materials has increased and is becoming common in the polymer industry [72].

Cellulose, the most abundant biopolymer in nature, has promising uses as reinforcement of mechanical properties in polymeric bionanocomposites. Nanocellulose also has several advantages for the development of new materials: The mechanical properties and its nanometric dimensions, together with its abundance, renewability, and biodegradability, open a wide range of possible applications for biocomposites or pharmaceutical carriers [74,75].

Lignin draws attention as a valuable environmentally friendly and biodegradable raw natural resource. Lignin is the second most abundant organic substance in the world, estimated to represent 30% of the total biomass produced in the biosphere and is mostly produced by the paper pulping industry [37].

#### 2.1.1. Lignocellulosic Materials

The use of natural fibers expanded due to the higher prices for petroleum products but also supported by the acceptance among consumers who encourage the use of renewable and non-polluting raw and waste materials [12]. As reinforcements in bioplastics, lignocellulosic fibers are biodegradable, renewable, and widely available; moreover, they have low density, competitive specific mechanical properties, and a relatively low cost [76,77]. Various polyolefins, polylactic acid, and PVA have recently been reinforced using cellulosic fibers from by-products from the production process in industry.

For example, some authors have tested composite formulations with mechanical and physical properties improved compared with the neat polymer and with the advantage of a higher bio-based content [78]. Thus, polypropylene composites containing dried distillers’ grains (DDG—10, 2.4, and 2.2 wt %), a coproduct of corn ethanol industries, maleated polypropylene, and maleated ethylene-propylene diene monomer rubber as compatibilizers, had improved strength and modulus compared with the neat polypropylene. The DDG with irregular shapes, mostly in the form of flakes—the majority of the particles were between 0.25 and 2 mm in their smallest dimension —was used in the as-received form with no surface modification or size reduction treatment. [78].

It is known from many reported papers that incorporation of natural fibers from approximately 20 to 35 wt % in polymer composites is the most appropriate amount to achieve optimum technical and economic benefits and addition of more fiber does not lead to any significant enhancement in the mechanical properties of the fiber composites [79,80].

Thus, Yerba mate (*Ilex paraguariensis*) fibers (20, 30 and 40 wt %) were used as reinforcing filler in polypropylene composites in order to increase the mechanical properties of the composites. The residue, ground and sieved to obtain a fibrillar structure with diameters above 100 μm, was processed into PP in a single-screw extruder, then ground in a knife mill, oven dried, and reprocessed in a twin-screw extruder [81]. The study revealed that the tensile strength at break was highest for 30% yerba mate but with further reduction for higher amounts of Yerba mate fiber. The addition of the filler also introduced regions of poor interfacial adhesion and stress concentration in the composite due to the imperfection in filler dispersion (agglomeration) [81], which explains the lower flexural strength of the composites and a gradual reduction of the impact resistance with the increase of the Yerba mate fiber content. Similar results have been obtained by other authors: High density polyethylene composites filled with three kinds of shell fibers (peanut shell, rice husk nor walnut shell −30–70 wt %), [82] increased the creep resistance and decreased the impact strength of the three composites for all compositions studied [82], while the bending and tensile strength increased first with the fiber content, and then decreased [82].

The mechanical properties (tensile strength, Young’s modulus, flexural strength, and flexural modulus) of polylactic acid (PLA) composites reinforced with hybrid sisal and hemp fiber (30 wt %) were improved compared to neat PLA [83]. The dried hemp and sisal fiber (a by-product from the production process in industry, where the leaves of the plant are predominantly used) [79] were aligned and granulated into 4 mm lengths and further processed/blended through extrusion and injection molding [83]. Thus, it is possible to replace 30%–40% by mass of the matrix with fibers (a by-product from the production process in industry, where the leaves of the plant are predominantly used) [81].

#### 2.1.2. Lignin

Lignin, a natural polyphenol [38], is a major component of all plants and an enormous renewable material and is mainly produced as a side-product by the bioethanol and the paper industry during the cellulose extraction [38].

As a thermoplastic polymer with high-impact strength and heat-resistance, lignin can be compounded within a polymer matrix, enhancing the mechanical properties due to the relatively high rigidity of the 3D network structure of the lignin molecules [40]. Blending lignin with other biopolymer or synthetic polymer materials has been attractive because of its wide availability, good mechanical properties, and biodegradability, along with the diversity of potential modifications due to its chemical structure [84]. For example, solution-casted lignin-modified PVC membranes had improved hydrophilic and anti-fouling properties together with improved stability and durability after a 6-cycle oily wastewater treatment of PVC membranes [85].

**Figure 4:**
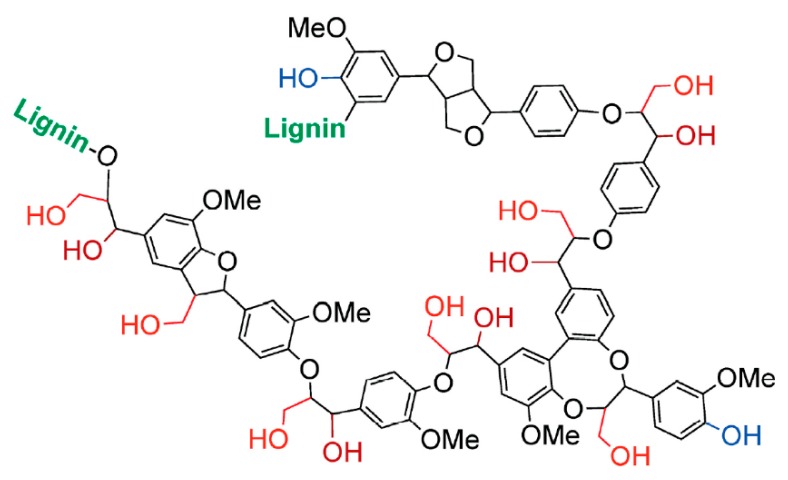
Structural representation of lignin (methyl groups abbreviated Me) ([86]).

Lignin is generally described as a highly cross-linked co-polymer containing phenyl-propanoid units linked together through a variety of C–C and C–O bonds, resulting in the presence of aliphatic hydroxyl and phenolic hydroxyl groups (Figure 4) [86]. Due to its numerous hydroxyl groups, the lignin molecule is relatively polar and generally presents poor compatibility with nonpolar polymers, such as polyolefins and polystyrene (or aromatic polymers) [38]. Lignin can enter only into weak dispersion interactions; thus, in the lignin/polyolefin systems limited compatibility is expected [38], which may be improved by using compatibilizers, reactive compatibilization [40], chemically modified lignin, or by adding a coupling agent [38]. Due to the strong self-interactions, lignin forms immiscible blends (0–60 vol % lignin) with ethylene-vinyl alcohol (EVOH) copolymers (0–76 mol % vinyl alcohol) as lignin was dispersed in the form of particles in the EVOH phase even at lignin contents as large as 60 vol %, in spite of the hydrogen bonds acting between the two components. Additionally, as the equilibrium thermodynamic factors are stronger than kinetic ones in the studied system, changing shear stresses during the melt (220 °C) blending process did not influence particle size much [87].

The hydroxyl content influences the compatibility between lignin and polyolefins: The less aliphatic hydroxyl content (0.4 mol/kg) of de-polymerized hydrolysis lignin (DHL) promotes better dispersion in polyethylene (PE) compared with de-polymerized kraft lignin (DKL) (0.7 mol/kg) [88].

Many studies have focused on the incorporation of lignin into natural biopolymers, such as starch, protein, cellulose, PLA, and PHB, to form bioplastics [89]. For example, the properties of the starch/lignin bio-nanocomposites, such as water uptake, water swelling, hydrolytic degradation, water vapor permeability, mechanical, and thermal properties, were enhanced significantly due to the lignin addition [90]. Lignin caused the rougher surface of the starch film, modified some structural properties, and improved thermal properties [91]. The addition of alkali lignin (AL) as a filler into the soy protein isolate (SPI) films increased the UV light absorption, improved tensile strength (TS) and thermal stability, as well as decreased the water vapor permeability of the films [92].

In addition, lignin plays an important part in antioxidant properties as a stabilizer because the phenolic hydroxyl groups can scavenge free radicals [93]. The antioxidant behavior of lignin stems from its inherent hindered phenolic structure, which facilitates lignin to work as a free radical scavenger [94]. For example, addition of 2.5 wt % of de-polymerized kraft lignin (DKL) or 5 wt % of de-polymerized hydrolysis lignin (DHL) to polyethylene (PE) attained the same level of antioxidant activity as the addition of 0.5 wt % of Irganox 1010 (a phenolic antioxidant—pentaerythritol tetrakis(3-(3,5-di-tert-butyl-4-hydroxyphenyl)propionate). The lower antioxidant activity compared to the commercial antioxidant, caused by the lower phenolic concentration, complex molecular feature, and higher hydrophilicity of the de-polymerized lignins, can be compensated for by the lower price of the de-polymerized lignins [88].

#### 2.1.3. Nanocellulose

Nanocellulose fibrils, which are the building blocks of plant cell [95] walls and give plants and trees their structural strength and stiffness, are a very interesting and well-studied nanoscale natural material that can potentially improve the properties of the polymers [96]. Many studies have already reported on use of nanocellulose in different forms for composite applications [70]. Cellulose nanomaterials are considered as a suitable solution to replace commonly used inorganic nanofillers [97] because they are widely available, renewable, and biodegradable [96]. Moreover, they possess unique valuable characteristics, such as a high specific strength, moduli (100–200 GPa), and specific surface area. Composites with nanocellulose nanoscale reinforcements have been proposed [98].

Regarding the nanocellulose, there some particularities compared with other artificial nanoparticles: In the case of carbon nanoparticles or nanoclays, the mechanical constraining of polymer chains [99], and surface/polymer interaction (with formation of layer of perturbed macromolecules around nanoparticles [100]) are supposed to be responsible for the beneficial effect of nanoparticles on the mechanical properties. In the case of nanocellulose, different mechanisms allow the nanocellulose to influence and enhance the polymer properties [101], i.e., the formation of a network of long nanoscale fibers [101]; the interaction between nanoparticles/nanofibrils, which is controlled not only by Van der Waals forces but also by strong hydrogen bonds [102,103]; and also the adhesion to the polymer matrix, which is dominated by hydrogen bonding [104,105]. Different to carbon nanoparticles, nanocellulose fibrils do not form clusters but percolation networks [106], which have a positive effect on the strength of the composites [107].

**Figure 5:**
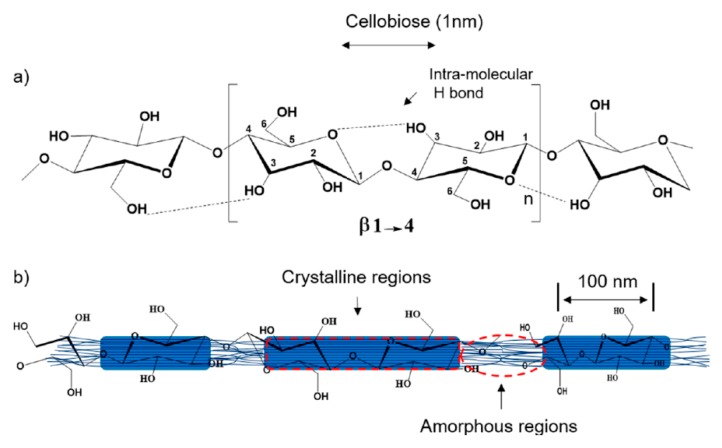
(**a**) Schematic of cellulose repeating unit with the β-(1,4)-glycosidic linkage; (**b**) hypothetical configuration of ordered (crystalline) and disordered (amorphous) regions in cellulose nanofibrils. ([108]).

Cellulose nanofibers are characterized by a short-rod-like shape less than 100 nm in diameter and several micrometers in length with ordered regions (Figure 5), induced by the linear nature of the cellulose polymers and the extensive intermolecular attractions between adjacent chains [108]. Cellulose nanofibers can be extracted from different natural sources [109] (wood or non-woody plants [110]), by mechanical treatment [111], acid hydrolysis (the most-used method) [112], or a combination of the two [113]. Acid hydrolysis can be assisted by ultrasonic treatment (sonication) [114,115] and enzymatic hydrolysis [116].

Acid hydrolysis is an easy and fast method to produce nanocellulose. A strong acid such as H2SO4 or HCl is commonly used to break the glycoside bonds in cellulose under controlled conditions (acid concentration, time, temperature, and acid/cellulose ratio) [117], a process which is stopped by dilution with water followed by washing/dialysis to remove free acid molecules and drying of the suspension to yield solid nano-cellulose [112]. Acid hydrolysis leads to agglomerated cellulose in the micrometer scale, which can be reduced to nanofibers in the nm scale by high pressure homogenization [118] due to the shear forces caused by the high velocity and pressure on the micro-suspension of cellulose nanofibers [119,120]. Using this procedure and high pressure homogenization, ginger cellulose nanofibers (GNF) (100–200 nm width and 2–3.5 μm length) were isolated and used (1% to 7% GNF) in bionanocomposites prepared by the solvent cast method in order to reinforce chitosan (CS) and polyvinyl alcohol (PVA). The best GNF content was 5% in terms of mechanical properties [120] due to the formation of hydrogen bonds between GNF and both CS and PVA. As CS has antibacterial activities over a wide range of bacteria [121] the chitosan containing GNF film had a higher inhibitory effect against *Bacillus cereus*, *Escherichia coli*, *Staphylococcus aureus*, and *Salmonella typhimurium* [120] because the bacterial cell wall can adhere to CS by electrostatic interaction [122], thereby changing the structure and permeability of the bacterial cell membrane [122].

Cellulose nanocrystals (CNC) were also successfully isolated from waste cotton cloth fibers using a mixed acid hydrolysis of the extracted cellulose (H2SO4 (98 wt %), HCl (37 wt %), and deionized water solution at a volume ratio of 3:1:11.) and subsequently used as fillers to reinforce a polylactic acid (PLA) matrix (0–0.7 wt % CNC) [123]. The PLA/CNC solvent cast films showed high crystallinity, tensile strength, and elasticity modulus at 0.1% and 0.3% CNC content [123]. However, at higher CNC content (0.7%), the CNCs were not well distributed in the polymer matrix, leading to minimal light transmittance, crystallization, tensile strength, elasticity modulus, and elongation at break [123]. Furthermore, the elongation at break of PLA/CNC composite films decreased with the increase in CNC content due to the restricted mobility of polymer macromolecules due to the increased film stiffness [123]. A significant decrease in light transmittance (from 67% to 53% at λ = 800 nm) was observed for PLA/CNC composite films when the CNC content increased from 0.1% to 0.7% [123]. The surface structure of the PLA/CNC composite films became increasingly compact with the higher CNC content [123] while considerable particle aggregation was observed on the surface of the composite film with the highest CNC content due to the hydrogen bond formation [123].

For effective use of cellulose fibers, good dispersion and adhesion between the fiber surface and resin are essential [124]. However, the OH groups on the surface of cellulose fibers make them highly hydrophilic [125,126], while most polymer resins are hydrophobic, which leads to poor dispersion and adhesion. Furthermore, the OH groups from nanocellulose result in strong hydrogen bonding leading to agglomeration of these fibrils during the drying process [127], making them difficult to re-disperse these fibrils even in water [128]. To address this, some authors followed a new approach in which chopped, loosely packed (i.e., low density) freeze-dried cellulose nanofibrils (CNF) networks (foams, i.e., bundles of CNFs) were used as “microsponges” that polymer resins can penetrate during melt processing, creating mechanical interlocking by hydrogen bonding among CNFs as a reinforcing mechanism, provided that the low packing density was achieved [124]. The dried PLA/CNF composites were mixed in chloroform and fed to a high-shear mixer and finally compression molded [124]. The use of CNFs (10–40 wt %) increased the tensile strength (up to 80%), elastic modulus (up to 200%), strain at break (75%), and toughness (220%) compared to the neat PLA resin [124]. Similar improvements in tensile strength and elastic modulus were observed when the PLA/CNF composite was tested as material for 3D-printing, although strain at break and toughness values dropped [124]. Still, the shear forces in the 3D-printing process [129] resulted in orientation and stretching of these CNF bundles in the printing direction, leading to further increases in stiffness and storage modulus. In other words, beneficial controlled directional stiffening of the manufactured parts can be attained with the printing process [124].

The polymer nanocomposites are traditionally constructed utilizing one nanomaterial as a filler [130]. There is also a possibility to tune the mechanical properties through the introduction of a second nanofiller (for example carbon nanotubes together with montmorillonites [131]). Tricomponent materials containing poly(vinyl alcohol) (PVA) and cellulose nanocrystals (CNC)/chitin nanofibers (ChNF) as filler (total filler content 5 wt %) [132] showed an overall increase in stiffness, tensile strength, and thermal stability [132]. Furthermore, CNC /ChNF mixtures at certain ratios were able to more effectively reinforce PVA better than CNC or ChNF alone [132]. Results showed certain ratios between cellulose and chitin had the largest moduli and tensile strength. The authors suggested this is the result of chitin nanofibers binding to themselves through hydrogen bonding and also creating a high strength network with the CNC [132]. The authors also suggested that above certain CNC loadings (1 wt %) agglomeration occurs between particles which generates weak points in the material, which could explain the relative decrease in mechanical properties with loadings higher than 1 wt % [132,133].

The mechanical behavior of the gelatin scaffolding material used in tissue regeneration can be improved by reinforcing it with TEMPO-oxidized cellulose nanofibrils and subsequent cross-linking [134]. By dehydrothermal crosslinking (with formation of zero-length cross-linking without any bridging molecule), both intra- and inter-molecular peptide bonds within gelatin and amidic bonds between gelatin and cellulose nanofibers were formed, which increased the mechanical stability of the scaffold. The crosslinking also decreased the degradation rate by hydrolysis [135] during the healing process in order to allow the replacement of the scaffold with newly formed tissue [136].

Not only the mechanical properties but also the barrier properties and water solubility can be improved by addition of cellulosic nanofibers (CNF) [137]. Starch naturally has poor moisture resistance [138], so the addition of CNFs is an effective way to reduce its moisture absorption, moisture sensitivity, and to improve the mechanical strength and stability of starch [137]. Due to the highly crystalline and hydrophobic character of cellulose in comparison to the starch molecule, CNFs are less hydrophilic than starch, making them effective to improve the barrier properties by reducing water vapor permeability [137]. The addition of CNFs introduced a tortuous path for water molecules to pass through [137]. However, additional amounts of CNFs might agglomerate and form a heterogeneous film, which in turn facilitates the water vapor permeation and holes in the film [137]. Cassava starch filled with chitosan/glycerol/gallic acid/CNFs (0.15, 0.5, 0.1, 0-0.1 g/g starch respectively) were prepared by a subcritical fluid system, which promotes both hydrolysis and cross-linking reactions between chitosan and starch to form a strong network [139]. The addition of CNFs reduced the film solubility in water significantly due to strong interactions between starch [140] and chitosan chains [141] with cellulose through hydrogen bonds in the film matrix [137]. This affinity or compatibility between CNFs and the starch matrix can be attributed to the chemical similarities of starch and cellulose, the nanoscale of the fibers, and the hydrogen bonds between CNFs and starch [137]. Due to the formation of hydrogen bonds between starch, chitosan, and CNFs, the gallic acid and chitosan addition to the CNF/starch also decreased the number of active −OH groups in starch and cellulose that promotes water absorption reducing the film water activity [137]. Because the addition of CNFs decreased the mobility of the starch and chitosan chains, the tensile strength increased, and elongation decreased considerably in CNF-reinforced films compared to cassava starch/chitosan/gallic acid film without CNF. Due to the cross-linking (by ester and electrostatic interactions) the addition of gallic acid and chitosan increased the tensile strength and decreased the elongation [137]. Moreover, due to the aggregation that occurred in films with 15 and 20 wt % of CNFs, the tensile strength did not increase above 17 MPa [137].

Table 2 summarizes the most recent examples of natural additives in polymers used as antioxidants, antibacterials, plasticizers, or used to increase degradability and thermal insulation. Besides lignin, natural fibers, plant extracts, and cellulose nanocrystals are presented, as well as other natural additives, such as chitosan, essential oils, and alginate.

### 2.2. Plant Extracts as Active Ingredients in Food Packaging Materials Based on Polysaccharide Matrices (Chitosan/Starch/Alginate)

**Figure 6:**
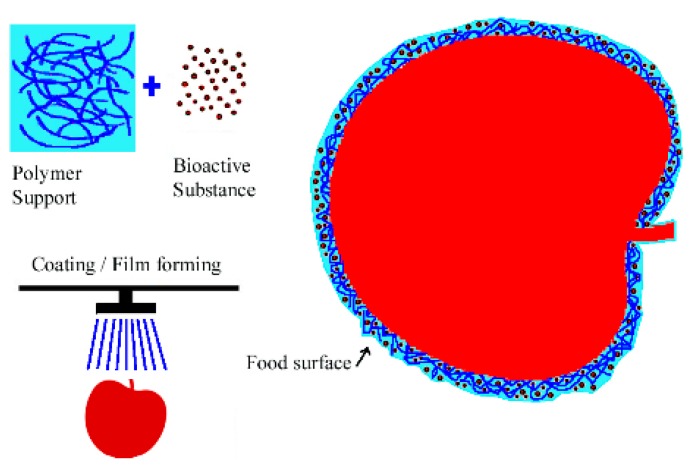
Basic representation of the edible coating forming process.

The need for better preservation of perishable food products, such as fruits, vegetables, or meat, has raised new postharvest preservation technologies, such as edible coatings, coating the solid package by electrospinning, UV irradiation, modified atmosphere packaging, and ozonation [150,151]. Edible coatings—a thin layer formed on the food surface to extend its shelf life—can preserve the properties and functionality of foods as they are easy to apply by spraying or immersion (Figure 6), and can be prepared with environmentally friendly materials [152].

There is great interest in biodegradable active composite packaging materials that release substances for the purpose of extending shelf life, by incorporation of active substances, such as antioxidants, antimicrobials, and antifungals, and which can enhance barrier, thermal, and mechanical properties [153]. Herbs, spices, agricultural waste as extracts, essential or cold-pressed oils contain bioactive compounds as thymol, carvacrol, tocopherols, benzoic acids, simple or functionalized phenolics and flavonoids, lignans, etc., which confer onto them activities and nutritional values. Representative examples of their structures can be found in References [154,155].

The mechanical and antibacterial properties of chitosan edible films were improved after the incorporation of gelatin and natural cinnamon essential oil [156]. Cassava starch food packaging films with antioxidant, UV-vis light barrier and pH-sensitive properties, increased water vapor permeability, and tensile strength were also obtained by addition of anthocyanin-rich bayberry extract (1 wt %) due to the hydrogen-binding interactions between BBE and starch [157]. Similarly, the incorporation of hydrolyzed cottonseed proteins into alginate films increased the thickness and water vapor permeability, the barrier properties to visible light, and the total phenolic content and the antioxidant activity with inhibitory effect against *Staphylococcus aureus*, *Colletotrichum gloeosporioides* and *Rhizopus oligosporus* [158].

Electrospun antibacterial and antifungal coatings form the base solid package: Antibacterial and antifungal active elements can be incorporated in chitosan matrices by electrospinning [159], for example, antibacterial polylactic acid/AgNPs/vitamin E nanofibers presented antioxidant activity during tests on fresh apple and apple juice due to reduced polyphenol oxidase activity, which make this materials a potential preservative packaging for fruits and juices [160]. The electrospun nanofibers containing low amounts of bioactive substances can be coated onto various substrates [161,162]. For example, polylactic acid (PLA) films coated by coaxial electrospinning with clove and argan oils and encapsulated into chitosan had higher antibacterial and antioxidant activity when clove oil and high molecular weight chitosan where used [163]. Polyethylene films coated with chitosan by electrospinning had good antimicrobial activity against Gram-positive (*Listeria monocytogenes*) or Gram-negative (*Escherichia coli, Salmonella typhymurium*) bacteria and the addition of vitamin E to the coatings improved the aspect, smell, pH, and total number of germs for minced poultry meat packaging [164,165].

#### 2.2.1. Chitosan/Starch/Alginate Containing Plant Extracts as Edible Food Packaging

Edible food packaging and coating materials are renewable and easily degradable that can enhance the shelf life of whole as well as fresh-cut fruits and vegetables by retarding physiological processes, such as respiration, degradation of cell walls, and transpiration, and also restrict microbial action [166]. They are generally obtained from polysaccharides such as starch, alginate, and chitosan [167].

Starch is the most abundant and commonly used renewable, biodegradable natural resource, with recognized flexibility, transparency, and film-forming ability, but with low mechanical and barrier properties that are usually improved by mixing the starch with other polymers (e.g., chitosan) [168]. For example, previous studies have reported that the chitosan–starch blends exhibited an improved barrier and mechanical properties as well as good antimicrobial effect [169,170]. Alginate is a naturally occurring polysaccharide can be used in coating for extending the shelf-life of fruit, vegetable, meat, poultry, and film preparation [171]. Chitosan is a good candidate for food packing material because of its film-forming ability, biodegradability, and satisfactory mechanical strength [172].

Alginate, starch, and chitosan are materials of choice for matrices used in a wide range of applications like pharmaceutics and medicine, active food packaging, and agriculture, to develop an environmentally friendly slow or controlled release systems for drugs, bioactive compounds, and respectively pesticides entrapment/encapsulation, and so on. This is because they alone or in binary and ternary combinations of them, or with other polymers, accomplish the main requirements necessary in these fields. They have a hydrophilic nature, are biocompatible, biodegradable, highly versatile, form non-toxic matrices for the protection of active ingredients (especially probiotic microorganisms and cells sensitive to heat, pH, dissolved oxygen) among other factors in which food is exposed to during processing and storage, offer economics and safety of processing methods, etc. These polymers are also presented as tasteless and odorless food additives. Chitosan and alginate exhibit mucoadhesion, immunogenicity, thickening properties, and the ability to form gels in the presence of multivalent ions, and are very good coating materials and show properties which allow their processing by simple one-stage processes (as the external gelation technique.). They are preferable for use in microparticles production for medicines [173,174] and pharmacy [175].

Microencapsulation is widely studied to protect microorganisms from acid environments, bile salts, and oxygen. This helps to maintain the microorganism’s viability during the product shelf life, which is a one of the major challenges to the food industry, since certain cultures are extremely sensitive to environmental factors such as acid and oxygen. Both maize starch and chitosan provided better protection of probiotics after exposure of the moist microparticles to simulated gastric and intestinal juice [176], demonstrating that a combination of alginate with starch improves the efficiency of different bacterial cells, particularly lactic acid-producing bacteria, due to the production of granules of good prebiotic structure and effect in the microcapsules.

A starch–chitosan–calcium alginate system showed the highest entrapment efficiency, drug/pesticide loading, and the slowest release rate, as well as an obvious slower degradation rate in soils than the commercially available formulation. [177,178]. The leaching of pesticides during the preparation of alginate beads has been improved by synthesizing bi-polymeric beads of alginate with other natural polysaccharides. An herbicide was encapsulated in chitosan and starch modified by cross-linking with alginate. The use of alginate helps bead formation and strengthens the structure of the matrices of the herbicide that was encapsulated in starch and chitosan beads reinforced with alginate. The addition of alginate improved matrix strength and prevented leakage of the encapsulated herbicide. [179].

Edible coatings could also be enriched with natural additives, such as antioxidant antibrowning [180] and antimicrobial agents, which in turn enhance the performance of the native edible coating material [181] as antioxidant activity, respiration rate, total phenolic retention, retention of ascorbic acid (AA), UV protection, etc.

The low antioxidant ability of chitosan [182] is usually improved by mixing chitosan with high antioxidant natural extracts (for example, tomato plant extract [183,184], leaf and seed extracts of *Pistacia terebinthus* [185], *Ficus hirta* fruits extract [186], flax seed mucilage extract [187], and *Laurus nobilis* extract [188]). Alginate and chitosan coatings containing olive leave extract were also found to improve the TP content and antioxidant activity of Sweet Cherries (*Prunus avium* L.). Alginate coatings containing *Fircus hirta* fruit extract enhanced the antioxidant activity and TP content in mandarin [189], and an edible coating from green tea extract and chitosan preserved the quality of strawberry (*Fragaria vesca* L.). Pomegranate peel extract (PPE) incorporated in chitosan had better retention of total phenol (TP), flavonoid content, antioxidant activity in terms of DPPH, and ripening index compared with pure chitosan, and also better than the corresponding alginate-based samples [190]. Similarly, blueberries coated with chitosan/ blueberry leaf extract (BLE) had higher TP than those coated with chitosan alone [191].

Respiration rate: One important function of edible films is to act as gas barriers and to create a biodegradable semi-permeable barrier protection and an internal modified atmosphere during the storage [192]. The application of various coating treatments could delay the rise in the respiration rate of the samples by causing changes in CO_2_ production and utilization of O_2_ [190]. A similar effect on retarding the respiration rate was found for mandarins coated with alginate containing *Fircus hirta* fruit extract [189] and avocado coated with chitosan containing moringa extract [193]. This impact of plant extracts was attributed to their antimicrobial activity that enhanced the barrier properties of the coating and restricted the gas diffusion [190] as other authors found similar effects for chitosan coatings incorporating natamycin or nisin, i.e., reduced the O_2_ consumption of the fruit, delayed changes of pH, and lower CO_2_ production during the storage compared to fruits coated with pure chitosan [194].

Total phenolic retention: Under oxidative stress, the total phenolic (TP) levels tend to decrease during storage period [195,196] and in the uncoated samples may be attributed to the higher respiration rate resulting in the breakdown of total phenols [197]. To slow down the deterioration events, such as respiration and moisture loss, ready-to-eat products should be protected with an edible film that modifies the internal atmosphere, resulting in slowing down the metabolism in fresh product [198].

Coating treatments could delay the declining of the total phenolic content, flavonoids, and anthocyanins content and antioxidant activity in various fruits as was found for strawberries coated with alginate containing carvacrol and methyl cinnamate (natural antimicrobials) [199], walnut kernel coated with sodium alginate coating containing pomegranate peel extract [200], and strawberries coated with chitosan containing peony extracts [201].

Retention of ascorbic acid (AA): Ascorbic acid prevents the oxidative damage in fruits and vegetables by scavenging the free radicals of the hydroxyl group, superoxide anion, and hydrogen peroxide through the ascorbate peroxidase reaction [202]. The ascorbic acid in fruits decreases continuously during the storage time [203]. In this respect, chitosan and alginate coatings enriched with PPE could retard the oxidation process in guava by maintaining higher levels of AA than the control during storage and, thereby, prevented ageing and resulting in lower oxygen permeability followed by reduction in the enzyme activity and, thereby, resulting in the reduction of AA oxidation [190].

UV protection: Food products are sensitive to various light wavelengths. Protection against light is an important and critical property of food packaging material, since light can accelerate food degradation and oxidation, resulting in nutrients and bioactive compounds destruction, forming off odors and flavors, food color loss, as well as the formation of toxic substances degradation and oxidation reactions [204].

Chitosan–plant extract films could effectively protect food against UV/visible radiation, for example, transmittance of chitosan–black soybean seed coat extract (BSSCE) was nearly in the 200–320 nm interval [205] and chitosan films containing aqueous hibiscus extract (HAE) exhibited transmittance below 10% in the 200–600 nm range [204], which was explained by the presence of several compounds in the plant extract, such as flavonoids, anthocyanin, phenolic acids, etc., which contains double bonds and could absorbs in UV/visible radiation [206].

#### 2.2.2. Phenols from Plant Extracts as pH-Sensitive Indicators of Chitosan/Starch/Alginate Matrices

Intelligent packaging combines benefits from active (antimicrobial, antioxidant, etc.) and intelligent packaging (sensing and sharing information about the condition of packaged products) [207]. Food spoilage is associated with pH change, which enables the use of alternative, inexpensive, natural-based colorimetric pH indicators in the form of labels or tags. These pH-sensitive indicators could react with the non-neutral volatile gases that were generated from foods during spoilage and are composed of natural and safe pH sensing pigments, such as anthocyanins and curcumin and a dye carrier/(solid matrix) [208]. Both ingredients have to be non-toxic, meet the food safety requirements, and be stable at the applied pH [209].

**Figure 7:**
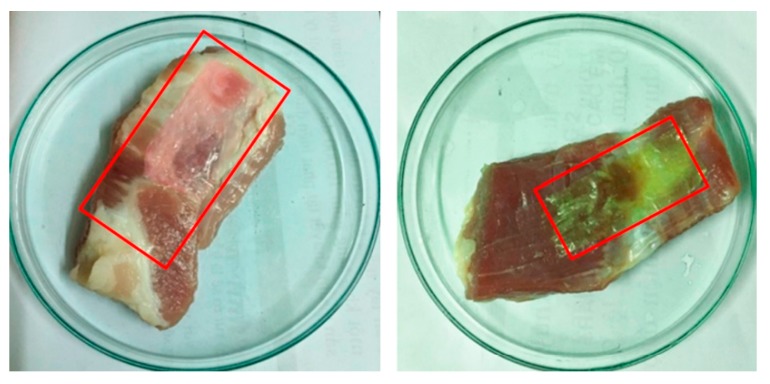
Color change of poly(vinyl alcohol)/chitosan/anthocyanin films in contact with raw pork belly slices exposed to ambient air for 12 h (left) and 24 h (right). After 12 h, the wrapping film becomes pink indicating an acidic condition near pH 5–6 on the surface of the pork slices. With further exposure for another 8 h, the pork meat turned dark-brown and softer, while the pH indicative film became yellowish with pale green, corresponding to a slightly alkaline range ([221]).

Anthocyanins are natural-based, non-toxic, water soluble pigments, extracted from different plants that provide the purple, blue, and red color of many plants [208]. Anthocyanins possess excellent antioxidant potential [210,211,212], which can be released from packaging films [213] for extending shelf life of food, anti-inflammatory [214], and anticarcinogenic [215] activities. Moreover, the colors of anthocyanins are sensitive to pH changes due to their structural transformations [216,217]. Thus, films incorporated with anthocyanins can be used as antioxidant, intelligent food packaging that can monitor the food quality (as colorimetric indicators) [218] because food spoilage is frequently associated with pH change (Figure 7) [219].

For example, by changing pH from 7 to 3, different color variations (from brown to bright red) were observed in chitosan films containing black soybean seed coat extract (BSSCE) rich in various anthocyanins while the plain chitosan film was colorless and did not respond to pH changes [205]. Similarly, the color of purple-fleshed sweet potato extract (PSPE) encapsulated into the chitosan matrix could change the color with pH from pink-red (pH 3–6) to purple-brown (pH 7–8) to finally greenish-green (pH 9–10) (depending on the incorporated amount of PSPE) [220]. Likewise, aqueous hibiscus extract (HAE) encapsulated in chitosan changed color under different pH conditions: brown to lightly greenish tones were observed at pH values ranging from 5 to 8 while yellow color was observed at pH 13.0 [204].

Anthocyanins, which are phenolic compounds with hydroxyl groups used as an indicator dye [221] could reduce the solubility of chitosan in distilled water [205] as they proved to support chitosan hydrogel formation with reduced water absorption and solubility [222]. Migration tests that showed that anthocyanins from chokeberry extract are both chemically bonded and physically immobilized in the chitosan matrix due to the interactions between the phenolic components and the hydroxyl and free amine groups of chitosan [209]. This is an important feature for the edible coatings: Applying the coating in humid conditions but not liquid media, or on the surface of the product, would not lead to leakage of the dye [209] while the coatings kept their swelling ability, which still provided efficient pH sensing in the bulk material [209].

#### 2.2.3. Plant Extracts Incorporated As Antioxidants in Chitosan/Starch/Alginate Matrices

Oxidative reactions in food as a deteriorative process promote the discoloration and the development of rancidity and off-flavors [223], which is usually overcome by using synthetic antioxidants with suspicious health properties [224]. Currently, natural antioxidants are studied because they are considered to be safe and due to the high consumer acceptance and willingness to buy these products [225]. Incorporation of natural antioxidants into packaging material (active packaging) [226] can be more effective than adding high levels of additives directly into the food [227].

Plant-derived phenolic compounds are natural antioxidants [228] that can scavenge reactive oxygen species by inhibiting the oxidation of low-density lipoproteins [229], which makes them substitutes for synthetic antioxidants in the food industry [230,231,232,233]. These polyphenols could be incorporated [234] within a film or coating applied to the food [235], which could release the antioxidant into the product or act on its surface, limiting the oxidative reactions of food components [236]. Aromatic herbs containing polyphenols have been traditionally used as healthy food ingredients [237,238,239,240] obtained through an aqueous extraction [241,242] process.

The direct addition of polyphenols to food is limited by their relatively rapid depletion [243]. The combined use of natural antioxidants and packaging materials increases the effectiveness of the antioxidants due to the protective effect of the chitosan matrix against polyphenol oxidation during storage of the films [244] and extending their action during a longer time [245,246].

For example, the use of a chitosan coating enriched with moringa leaf extract on avocado [193] and alginate coating enriched with grape seed extract on grapes [247] have demonstrated improvement in their overall quality. Similar enhancements in antioxidant activity were achieved when grape pomace extract [248], maqui berry extract [249], thinned young apple [250], peanut skin (EPS), and pink pepper residue (EPP) extract [251] polyphenols were incorporated into a chitosan film while the chitosan film itself showed very low antioxidant activity [252] due to the absence of antioxidants along with the presence of catalysts of the lipid oxidation (light, technological treatment, presence of salt and unsaturated lipids) [251]. Blueberry leaf extracts (BLE) incorporated into a chitosan coating could maintain higher radical scavenging activity (RSA) of fresh blueberries during refrigerated storage in the first days of storage, but after three days the samples had lower RSA than the control and chitosan coating alone [191].

As chitosan interacted with the polyphenols to a greater extent than starch, it is reported that the fastest delivery rate and the higher delivery ratio of thyme extract polyphenols [253] were when starch is used as the matrix, which is explained by the high solubility of starch matrix, without the crosslinking effect with the phenolic compounds [253].

The peroxide value (the quantity of the total primary oxidation products present in edible oils [254]) of rainbow trout coated by dipping with films containing chitosan and different concentrations of pomegranate peel extract (PPE), was significantly lower compared to the control [255] during storage time due to the proper antioxidant effect of the PPE, which inhibited superoxide hydroxyl and peroxyl, which finally lead to the oxidation of fats [256]. However, even though the best performance to prevent the oxidation of fats and proteins and also antimicrobial efficacy was observed for higher plant extract content, due to its undesirable color, the chitosan combined with 2% PPE was preferred [255].

#### 2.2.4. Phenols from Plant Extracts as Crosslinkers for the Chitosan/Starch/Alginate Matrices

Swelling ability is beneficial for the release of active substances and also in the case of indicator labels [257] because the aqueous media with the changed pH can penetrate into the bulk matrix, which enhances the effectiveness of the pH sensing and also controls the release. In the same time, dissolution and degradation are undesired [258]. For chitosan films containing active substances it is important to reduce its solubility (e.g., via cross-linking) [259], thus preventing the indicator label/active substances from dissolving when pH is lowered due to food spoilage as the pH affects the swelling and release behavior of the active substances [260].

In order to replace toxic chemical cross-linkers for hydrogel formation, such as glutaraldehyde [261] or epichlorohydrin [262], edible chitosan films with reduced solubility can be obtained by using cross-linking agents extracted from natural plants [263].

Phenolic components obtained from different kinds of plants can interact (intermolecular interactions) with hydroxyl and free amine groups of [264,265] that reduce the solubility of chitosan films: by hydrogen-bonding, electrostatic attraction (ionic complexations in acidic conditions) [266] between the anthocyanins and chitosan, and even through ester linkage [253,267]. For example, a small amount of purple-fleshed sweet potato extract (PSPE) (5 wt % on chitosan basis) could establish physical interactions with chitosan molecules and act as bridges among different chitosan chains [205,220].

Thus, the incorporation of pure phenolics or phenolic-rich plant extracts [42,268], in addition to the increased antioxidant ability, can greatly enhance the physical property (by crosslinking through non covalent bonds) of chitosan film (thinned young apple polyphenols [250], thyme extract [244], and protocatechuic acid—a potent antioxidant agent found in fruits and vegetables [269]), leading to a more rigid and compact chitosan matrix and improving the film physical properties [244].

Tannic acid is a polyphenol naturally found in some green leaves, which exhibits antioxidant properties due to its multiple phenolic groups which can help crosslinking chitosan films [270], a process facilitated by exposure to high temperatures (100 °C) [271]. Thyme-extract polyphenols have been also used as crosslinkers [253,272].

The interactions of chitosan with phenolic substances through non-covalent bonds were proved by FTIR analysis, for example, for chitosan films containing black soybean seed coat extract (BSSCE) [205].

The IR spectra evidenced that the incorporation of the phenolic substances into the chitosan led to the presence of a new absorption band appearing at 1707 cm^−1^ [273] (1715 cm^−1^ [267]), or 1660 cm^−1^ [267], which became more pronounced by increasing the extract content [207] and was attributed to an ester linkage [267,274] (C=O stretching [273]).

For the chitosan purple-fleshed sweet potato extract (PSPE) films, the band around 3290 cm^−1^ broadened, while the band of N-H bending (1553 cm^−1^) gradually decreased with the increase of PSPE amount [220]. The absorptions at 3500–3000 cm^−1^ reflecting the stretching vibration of hydrogen bonds between the lattice hydroxyls and organic groups were stronger in pure chitosan film compared to those incorporated with pomegranate rind powder extract (PRP) [273] and was shifted to lower frequencies in chitosan/pomegranate rind powder extract (PRP) blended films [273]. The peaks at 1561 cm^−1^ (N–H band) and 1422 cm^−1^ (C–H band) (characteristic peaks of chitosan) flattened with incorporating PRP [273]. Other authors reported a gradual decrease of the 1553 cm^−1^ band (N–H bending) with the increase in PSPE amount [220].

All these observations suggest interactions [273] between the amine groups of chitosan (hydroxyl/amino groups [220]) and the acid groups (e.g., hydroxyl groups [220]) of the phenolic compounds [267].

Similarly, the color data regarding the films with different concentrations of phenolic compounds measured at different times proved that there are interactions between the chitosan and the phenolic compounds present in the extract [267].

The beneficial effects of the interactions (crosslinking) of phenolic substances with the chitosan/starch/alginate matrices are 1) delaying/controlling the release of the active substances: The incorporation of tannic acid as crosslinking agent into chitosan films delayed the thyme extract polyphenols release rate in water and ethanol aqueous solutions, and also reduced the average water content of chitosan–starch films without affecting their solubility [253]; 2) enhancing the mechanical behavior: When mixed with chitosan, the polyphenols from thyme extract interacted with the chitosan chains, acting as crosslinkers and enhancing the tensile behavior of the films. The opposite effect was observed when incorporated into the starch matrix [244]. The supplemental addition of tannic acid to the chitosan-based films also produced a further significant increase in the elastic modulus, tensile strength, and lower elongation at break due to the crosslinking effect [244].

Similar improvements were reported for chitosan films incorporating murta leaf extracts, rich in polyphenolic compounds [275]. Higher tensile strength as compared to plain chitosan film were found in chitosan green tea extract [276]. However other authors reported that the tensile strength of chitosan films containing anthocyanin-rich purple-fleshed sweet potato extract (PSPE) was not very different from those of pure chitosan films but decreased the elongation at break [220,252]. However, a significant reduction in the tensile strength was detected when PSPE amount increased to 10 wt % [220]. The incorporation of PSPE also caused significant decreases in the diffraction peak intensities of chitosan with the increase in PSPE amount due to newly formed intermolecular hydrogen bonds between the chitosan chains and anthocyanins in PSPE [220].

3) Decreased water barrier properties (decreased water vapor permeability (WVP)): Tannic acid (TA) was reported to decrease water vapor permeability (WVP) due to the same crosslinking effect for chitosan [271]. However, other authors evidenced the water plasticization effect on the polymeric matrix which seems to mitigate the crosslinking effect of TA, as pure chitosan and chitosan: TA films had greater values of water vapor WVP and oxygen permeabilities than the starch films while the chitosan:starch films exhibited intermediate behavior [244].

In addition to the mentioned crosslinking effect, other authors attribute the enhanced water vapor barrier property of chitosan films incorporating black soybean seed coat extract (BSSCE) to the bulky aromatic and pyrylium rings in the skeleton of anthocyanins that could obstruct the inner networks of chitosan–black soybean seed coat extract (BSSCE) films and reduce water vapor affinity of films [205].

The lower WVP of the films incorporated with different plant extracts may be also due to the hydrogen and covalent interactions between the chitosan network and polyphenolic compounds, which reduce the availability of the hydrophilic groups [277] and lead subsequently to a decrease in the affinity of chitosan film towards water molecules [252]. Similar decrease in WVP values were reported when chitosan film was incorporated with apple polyphenol. [250]. However, when the amount of the plant is too high (for example 15 wt % for purple-fleshed sweet potato extract (PSPE)), the dispersion of the extract destroyed the dense and compact structure of film, resulting in the increase of WVP [220].

4) Increased stability under different pH in aqueous media: The stability under different pH in aqueous media is an important requirement for the applicability of the edible coated films. The water uptake of chitosan was remarkably reduced by the presence of chokeberry pomace extract (AEX): the modified chitosan films were stable and did not break apart during the swelling tests even under acidic pH due to the interactions between chitosan network and polyphenolic compounds which reduce the availability of the hydrophilic groups [209].

5) Decreased moisture content: Due to this interaction, which greatly limited water–chitosan intermolecular interactions, the moisture contents in the chitosan films decreased with the addition of the polyphenols from plants extract. [220]. Similarly, chitosan–black soybean seed coat extract (BSSCE) films [205] or chitosan/aqueous hibiscus extract films [204] presented lower moisture contents in comparison to pure chitosan film. The opposite effect was observed for starch and gelatin films: The addition of the aqueous hibiscus extract promoted a significant increase in water content [204].

## 3. Conclusions

Plants provide materials which can be used as additives into polymeric materials, such as lignocellulosic fibers, nano-cellulose, or lignin, as well as plant extracts containing bioactive phenolic and flavonoid compounds used in the food packaging area. The lignocellulosic materials, lignin and nano-cellulose, can be used as reinforcements in various polymer matrices. As active ingredients in food packaging materials, based on chitosan/starch/alginate, the plant extracts can decrease the respiration rate and can delay the declining of the total phenolic content, flavonoids, anthocyanin content, and antioxidant activity in various fruits, as well as provide UV protection and can act as crosslinkers for the chitosan/starch/alginate matrices.

## Figures and Tables

**Table 1:** Natural compounds (polymers and natural extracts) used as components/additives in new polymeric materials together with their methods of preparation and applications.

Source and Compound		Obtaining Method	Mixing Method	Application
*Plant Sources*				
**Polysaccharides**				
	Cellulose; used as pulp, nanocrystals, nanofibers and fibers	Cellulose can be isolated using a combination of chemical and mechanical treatments like ultrasonication combined with chemical pretreatments, high shear homogenization coupled with acid hydrolysis and steam explosion, etc. [13].	Extrusion (for example in polypropylene composites [14]), reactive extrusion [15].	Reinforcement in polymer composites [14,16,17,18].
	Starch	Starch is extracted from seeds, roots and tubers, by wet grinding, washing, sieving and drying [19].	Extrusion, injection molding, film casting [20], reactive extrusion [15]. For incorporating starch in plastics, commercialized technologies were developed to overcome the moisture sensitivity and inferior mechanical properties of starch [21].	As a filler in biodegradable food packaging materials [22,23,24] or in plastic films can improve the biodegradability [25].
	Pectin	Extracted using acids and enzymes [26].	Extrusion (for example in polyvinyl alcohol composites) [27].	Antimicrobial packaging materials [28].
**Proteins**				
	Soy Protein, hydrolyzed proteins (wheat gluten, wheat gliadin), zein, polypeptides	- Alkaline extraction followed by protein precipitation at isoelectric pH; - protein extraction with salt solution, followed by precipitation from a salt extract by ultrafiltration, diafiltration membranes or dilution in cold water (micellization) [29]; and - novel techniques, such as ultrasound assisted extraction, enzyme-assisted extraction in the form of proteases and/or carbohydrolases [29].	Extrusion foaming [30], reactive extrusion [15].	Reinforcement in polymer composites [31,32]. Polypeptides: Reinforcement in polymer composites [33]. Food packaging applications [34] or incorporated as a reinforcement in films with enhanced barrier properties [35] (zein). Mixing different proteins with polysaccharides is an effective way to improve barrier and mechanical properties of protein- polysaccharides films [36].
	**Lignins**	Industrially, lignin is isolated from cellulosic fibers by chemical treatment, which breaks down lignin–carbohydrate complexes. During this process, partial depolymerization of the complex lignin macromolecules occurs along with re-polymerization (condensation) which may alter the native lignin structure [37]. The paper pulping process (lignin extraction from lignocellulosic biomass) which produces industrial lignin as a byproduct [37] may include chemical methods [38], such as - Kraft process which uses a mixture of Na_2_S and NaOH (White Liquor) at high temperature (150–180 °C), - sulfite process which employs sulfite or bisulfite to digest biomass, - organosolv pretreatment of lignocellulose which involves a biomass extraction in a mixture of solvent (ethanol being the most common) and water under high pressure [39], - single pot soda cooking pre-treatment for extracting lignin and isolate cellulose nanofibrils simultaneously [13].	The methods of blending lignin with thermoplastic polymers (natural or synthetic - as polyethylene (PE), polypropylene (PP), polyvinyl chloride (PVC), polymethylmethacrylate (PMMA), polyvinyl alcohol (PVA), ethylene-vinyl acetate copolymer (EVA), polyester, starch, and protein) include melt-blending (extrusion, compression, injection, and blow-molding) and solution mixing [40].	Lignin as reinforcer/fillers in thermoplastic polymers improved mechanical properties, decreased water absorption, antioxidant effect due to the phenols in the lignin structure [41], improved water resistance, and thermal stability of the natural polymers such as starch or proteins. [40].
	**Polyphenols** **Plant Extracts** **Essential Oils**	The most commonly applied methods for the extraction of polyphenols uses water in combination with organic solvents (acetone, ethanol, methanol, ethyl acetate) as per the type of polyphenols present in the plant [42].	Blending methods to circumvent the loss of the volatile compounds: - melt blending requires the addition of the active compound in a later stage of the mixing after the polymer is melted, low melting temperature and decreased mixing time [43]. - dispersion/dissolution of the polymer and all active components in a common solvent that is subsequently evaporated (solution casting technique)—method that can also be used as a coating technique by casting the dissolution onto the particular surface [43]. - novel method which involves electrospinning/electrospraying the polymer/active component solution—the advantage of faster solvent evaporation compared with the solution casting technique with the possibility to encapsulate volatile compounds into polymeric fibers/particles.	Plant extracts and essential oils [44,45] are mainly used as antioxidant and antibacterial agents due to the components present in essential oils (eugenol, eugenyl acetate, carvacrol, cinnamaldehyde, thymol, squalene, rosmarinic acid, tyrosol, β-caryophyllene [46]) and plant extracts (isoprenylflavones, flavonone phytoalexins, isoflavonoids, monomeric polyphenols, epicatechin, epicatechin gallate, epigallocatechin gallate, terpenes, alkaloids) [47]. The minimal inhibitory concentration of an antimicrobial agent is the lowest (i.e., minimal) concentration of the antimicrobial agent that inhibits a given bacterial isolate from multiplying and producing visible growth in the test system. For example, in ethanol, thyme, clove and tea tree essential oils had approximately 1, 12, 25 v/v % MIC against *Staphylococcus aureus* and 1, 3, 12 v/v % against *Escherichia coli* [48].
*Animal Sources*				
**Polysaccharides**				
	Chitin	Isolation of chitin from crustaceans, such as crayfish, crab, shrimp, and other organisms such as fungi [49], by deproteinization with alkaline treatment at high temperatures, and demineralization with dilute hydrochloric acid [50].	Chitin nanocrystals and nanofibers were added by melt-mixing as fillers into thermoplastic starch-based biocomposites [51]. Also, chitin nanofibers were added in molten PLA by extrusion [52].	Reinforcement in polymer composites [52,53].
	Chitosan	By chitin N-deacetylation [50,54].	Solvent blending [55,56], extrusion blending and reactive extrusion blending [57] as chitosan may be heated up to temperatures below its glass transition temperature without affecting its physicochemical properties [58].	Polymer composites (polyvinyl chloride, polyurethane) with antibacterial properties [59,60]. Reinforcement in polymer composites [54].
**Proteins**				
	Silk/Wool		- In thermoplastics: melt mixing, single/twin screw extruder, and compression molding - In thermosets: vacuum assisted transfer molding, vacuum bag resin transfer molding and vacuum-assisted resin-infused repairing [12].	Reinforcement in polymer composites [10,61].
	Collagen/hyaluronic acid	Hyaluronic acid it is mainly produced via streptococcal fermentation. Recently the production of hyaluronic acid via recombinant systems was studied due to the avoidance of potential toxins [49]. - Collagen can be basically obtained from the slaughter of pork and beef by chemical hydrolysis and enzymatic hydrolysis [62].		Bioactive composite scaffolds for bone tissue engineering [63,64].
*Mineral Source -* Clays/Nanoclays				
	Natural clays: e.g., montmorillonite, hectorite, sepiolite, laponite, saponite, bentonite, kaolinite,	Relatively simple techniques are used in industrial processing for separation and purification of natural clays: decomposition of carbonates, dissolution of (hydr)oxides, oxidation of organic material, dissolution of silica, dialysis, and fractionation. [65].	Polymer–nanoclay nanocomposites may be prepared by melt or solution blending, with partially exfoliated clays, in situ polymerization, and melt intercalation by conventional polymer extrusion process, microwave and ultrasound irradiation [66].	Nanoclays used as fillers in various polymer matrices enhancing mechanical properties of the polymer matrix [67]. In biomedical field: - nanoclays as fillers in chitosan poli e-caprolactone poly-ethylene glycol poly(2-hydroxyethyl methacrylate) for drug delivery applications, as reinforcements for PMMA composites for bone cement applications or implants with improved bioactivity and mechanical properties or incorporated to polysaccharide hydrogels that can support cell proliferation (chitosan, gellan gum) [68].

**Table 2:** Various natural additives used in polymers.

Matrix	Additive (content)	Mixing/Preparation Method	Role, Change in Properties/Observations	Ref.
**Antibacterial/Antioxidant Plastics**				
PVC-based composites with self-sterilizing and antibacterial activity against *S. aureus* (functional antibacterial plastic).	Chitosan (wt % 0–40).	The mix was melt-compounded in an internal mixer at 150 °C.	Chitosan addition increased Young’s modulus evidencing a good CS–PVC interaction. Chitosan addition had no negative impact on thermal stability of the PVC composites which allows for possibility of producing composites by with thermo-mechanical processes, without risk of thermal decomposition.	[59]
Biodegradable polymer fiber nets of poly (lactic acid) (PLA)/poly (butylene adipateco-terephthalate) (PBAT) (60:40). Packaging material for fruit and vegetables preservation.	Pine essential oil (10%–20%). Some formulations were additionally coated with chitosan (1%).	Extruded biodegradable polymer.	With essential oil addition increased plasticity (at 10% Pine EO), elongation at break and decreased Young’s modulus. When chitosan was added as a coating, stiffening of the fiber was observed.	[142]
PLA-based composites for the packaging industry.	Water-soluble extracts (2%; 10%; 20%; 30%) from banana pseudo–stems.	Solution blending, casting and thermocompression.	Water-soluble extracts acted as a plasticizer on PLA (Tg decrease) and has slightly positive influence on its stiffness in the glassy state, whereas the drawability remained fairly acceptable when PLA-based materials where drawn at 75 °C above Tg.	[143]
Antimicrobial PLA films for food packaging with low silver release.	Alginate microbeads obtained by electrostatic extrusion (200 μm) with incorporated AgNPs (1.5 wt % Ag; 3 wt % alginate).	Solvent casting	PLA matrix acted as a diffusion barrier so that the released silver in water after 10 days was within the prescribed limit of 0.05 mg kg^−1^ while the films induced inhibitory effects against *Staphylococcus aureus*.	[144]
Poly caprolactone (PCL) nano fibrous mat with antioxidant activity for antimicrobial wound dressings.	Extract of medicinal plant *Clerodendrum phlomidis*.	Electrospinning	The plant extract conferred antibacterial activity and increased in wettability of the PCL fibers without affecting their mechanical properties.	[145]
Polyethylene oxide (PEO) These results will recommend these films a potential candidate in electrochemical and photoelectrical devices.	Starch (30 wt %) doped with various concentrations of gold nanoparticles (Au NPs)	Solvent casting	Differential scanning calorimetry (DSC) measurement indicated miscibility between the two polymers. Found electrical conductivity increased as Au NPs content increased. The miscibility between PEO and starch could be due to the oxygen atoms of PEO interacting through hydrogen H-bonds between the hydroxyl groups of starch. DSC revealed that the thermal stability of the blend polymer decreased after addition of the nanofiller.	[146]
Poly(lactic acid), PLA. The low cost and toxicological impact make cardanol a valid alternative to the plasticizer PEG.	Cardanol derived plasticizers (10%, 20% and 30%); three different plasticizers were used: neat cardanol, cardanol acetate (CA), and epoxidized cardanol acetate (ECA) were used, at contents ranging between 10% and 30%.	Mixing PLA, pre-dried at 70 °C for 24 h, and different amounts of plasticizers (10%, 20%, and 30%) for 15 min at 190 °C in a HAAKE RHEOMIX 600\610 mixer, with a rotor speed of 60 rpm.	PLA plasticized by cardanol derivatives showed lower modulus than PEG plasticized PLA. The tensile modulus of plasticized PLA was correlated to the evolution of glass transition temperature and degree of crystallinity. At low plasticizers content, the modulus of PLA decreased as the glass transition temperature decreased, due to a better miscibility of the plasticizer with PLA. The opposite occurred at high plasticizer content; in this case, the higher modulus found for more compatible plasticizers were attributed to an increased crystallization kinetic.	[147]
PU polyurethane 3D-printed foams as thermal insulation, sound absorption or as damping materials.	Cork powder (1%, 3%, and 5% wt/wt).	The TPU powder was mixed with cork powder (1%, 3%, and 5% wt/wt) in the Retsch cross beater mill SK1 without sieves. Afterwards, the mixtures were left over night in an oven at 105 °C to remove moisture. The mixtures were then extruded in a Felfil Evo Colours extruder using 4 rpm at 210 °C to produce the 3D printable filaments.	3D-printed PU polyurethane composite foams for thermal applications with enhanced mechanical properties. Due to the presence of cork as well as to the presence of voids the resulting foams presented lower density, lower thermal conductivity and proved to be more flexible. The stiffness of the ensuing composites was also reduced but the elastomeric behavior of the 3D-printed foams produced may find applications that combine thermal insulation with damping properties. Yet, the use of cork did not affect the thermal stability of the composites. Cork is a well-known low thermal conductive material, which can further reduce the thermal conductivity of PU foams Besides their thermal insulation properties, their elastomeric behavior suggests that the 3D-printed foams produced may be used as thermal insulation, sound absorption or as damping materials.	[148]
Polyethylene/poly (lactic acid)/Degradable polymeric films	Chitosan (15 wt %) with and without poly (ethylene-g-maleic anhydride) (PEgMA) as compatibilizer.	Laboratory mixer-extruder. 145 °C and 155 °C for the screw barrel.	Polyethylene/poly (lactic acid)/chitosan films, with and without poly (ethylene-g-maleic anhydride) (PEgMA) as compatibilizer, were prepared by extrusion. It was demonstrated that blends of synthetic and natural polymers have a higher susceptibility to degradation in comparison to neat polyethylene and poly (lactic acid) films. Additionally, it is found that the incorporation of PEgMA into the extruded films apparently favored the polymer degradation, as it deduced from the fall of the mechanical properties when the films are exposed to accelerated weathering simulation.	[149]

## References

[1] Ambrogi V., Carfagna C., Cerruti P., Marturano V., Jasso-Gastinel C.F., Kenny J.M. (2017). Chapter 4: Additives in polymers. Modification of Polymer Properties.

[2] Scaffaro R., Maio A., Sutera F., Gulino E.F., Morreale M. (2019). Degradation and recycling of films based on biodegradable polymers: A short review. Polymers.

[3] Hasan M., Zhao J., Jiang Z. (2019). Micromanufacturing of composite materials: A review. Int. J. Extrem. Manuf..

[4] Chung D.D. (2017). Chapter 3-Polymer-Matrix Composites: Structure and Processing. Carbon Composites: Composites with Carbon Fibers, Nanofibers, and Nanotubes.

[5] dos Santos Rosa D., Lenz D.M., Chamy R. (2013). Chapter 16: Biocomposites: Influence of Matrix Nature and Additives on the Properties and Biodegradation Behaviour. Biodegradation: Engineering and Technology.

[6] Dhifi W., Bellili S., Jazi S., Bahloul N., Mnif W. (2016). Essential oils’ chemical characterization and investigation of some biological activities: A critical review. Medicines.

[7] Lim T.Y., Lim Y.Y., Yule C.M. (2017). Distribution and characterisation of phenolic compounds in Macaranga pruinosa and associated soils in a tropical peat swamp forest. J. Trop. For. Sci..

[8] Lalit R., Mayank P., Ankur K. (2018). Natural fibers and biopolymers characterization: A future potential composite material. Stroj. Cas. J. Mech. Eng..

[9] Jawaid M.H.P.S., Khalil H.A. (2011). Cellulosic/synthetic fibre reinforced polymer hybrid composites: A review. Carbohydr. Polym..

[10] Ramamoorthy S.K., Skrifvars M., Persson A. (2015). A review of natural fibers used in biocomposites: Plant, animal and regenerated cellulose fibers. Polym. Rev..

[11] Peças P., Carvalho H., Salman H., Leite M. (2018). Natural fibre composites and their applications: A review. J. Compos. Sci..

[12] Mochane M.J., Mokhena T.C., Mokhothu T.H., Mtibe A., Sadiku E.R., Ray S.S., Ibrahim I.D., Daramola O.O. (2019). Recent progress on natural fiber hybrid composites for advanced applications: A review. Express Polym. Lett..

[13] Ahuja D., Kaushik A., Singh M. (2018). Simultaneous extraction of lignin and cellulose nanofibrils from waste jute bags using one pot pre-treatment. Int. J. Biol. Macromol..

[14] Zander N.E., Park J.H., Boelter Z.R., Gillan M.A. (2019). Recycled Cellulose Polypropylene Composite Feedstocks for Material Extrusion Additive Manufacturing. ACS Omega.

[15] Formela K., Zedler Ł., Hejna A., Tercjak A. (2018). Reactive extrusion of bio-based polymer blends and composites-Current trends and future developments. Express Polym. Lett..

[16] Kaynak B., Spoerk M., Shirole A., Ziegler W., Sapkota J. (2018). Polypropylene/cellulose composites for material extrusion additive manufacturing. Macromol. Mater. Eng..

[17] Tolinski M., Tolinski M. (2015). Additives for Polyolefins: Getting the Most Out of Polypropylene, Polyethylene and TPO.

[18] Dufresne A. (2017). Cellulose nanomaterials as green nanoreinforcements for polymer nanocomposites. Philos. Trans. R. Soc. A Math. Phys. Eng. Sci..

[19] Dufresne A. (2012). Nanocellulose: From Nature to High. Performance Tailored Materials.

[20] Pérez-Pacheco E., Canto-Pinto J.C., Moo-Huchin V.M., Estrada-Mota I.A., Estrada-León R.J., Chel-Guerrero L., Poletto M. (2016). Thermoplastic Starch (TPS)-Cellulosic Fibers Composites: Mechanical Properties and Water Vapor Barrier: A Review (Chapter 5). Composites from Renewable and Sustainable Materials.

[21] Ayoub A.S., Lucia L.A., Ayoub A.S., Lucia L.A. (2017). Fundamental Science and Applications for Biomaterials (Chapter 2) Starch-Based Plastics (& 2.4.3). Introduction to Renewable Biomaterials: First Principles and Concepts.

[22] Sadeghizadeh-Yazdi J., Habibi M., Kamali A.A., Banaei M. (2019). Application of Edible and Biodegradable Starch-Based Films in Food Packaging: A Systematic Review and Meta-Analysis. Curr. Res. Nutr. Food Sci. J..

[23] Muller J., González-Martínez C., Chiralt A. (2017). Combination of poly (lactic) acid and starch for biodegradable food packaging. Materials.

[24] Kamilah H., Mahmood K., Poh L., Sulaiman S., Nafchi A., Fazilah A., Karim A. (2018). Food Packaging: Starch and Non-Starch Blend Films (Chapter 61). Encyclopedia of Polymer Applications.

[25] Glenn G.M., Orts W., Imam S., Chiou B.S., Wood D.F., Halley J., Avérous L. (2014). Starch plastic packaging and agriculture applications. Starch Polymers.

[26] Sandarani M.D.J.C. (2017). A review: Different extraction techniques of Pectin. J. Pharmacogn. Nat. Prod..

[27] Fishman M.L., Coffin D.R., Onwulata C.I., Willett J.L. (2006). Two stage extrusion of plasticized pectin/poly (vinyl alcohol) blends. Carbohyd. Polym..

[28] Liu L.S., Finkenstadt V.L., Liu C.K., Jin T., Fishman M.L., Hicks K.B. (2007). Preparation of poly (lactic acid) and pectin composite films intended for applications in antimicrobial packaging. J. Appl. Polym. Sci..

[29] Hadnađev M.S., Hadnađev-Dapčević T., Pojić M.M., Šarić B.M., Mišan A.Č., Jovanov P.T., Sakač M.B. (2017). Progress in vegetable proteins isolation techniques: A review. Food Feed Res..

[30] Gavin C., Lay M.C., Verbeek C.J. (2016). Extrusion foaming of protein-based thermoplastic and polyethylene blends. Proceedings of the AIP Conference, 31st International Conference of the Polymer Processing Society.

[31] DeButts B.L., Hanzly L.E., Barone J.R. (2018). Protein-polyisoprene rubber composites. J. Appl. Polym. Sci..

[32] Gu W., Liu X., Li F., Shi S.Q., Xia C., Zhou W., Zhang D., Gong S., Li J. (2019). Tough, strong, and biodegradable composite film with excellent UV barrier performance comprising soy protein isolate, hyperbranched polyester, and cardanol derivative. Green Chem..

[33] Li J., Li Y., Zhang J., Li G., Liu X., Li Z., Liu X., Han Y., Zhao Z. (2015). Nano polypeptide particles reinforced polymer composite fibers. ACS Appl. Mater. Interfaces.

[34] Altan A., Aytac Z., Uyar T. (2018). Carvacrol loaded electrospun fibrous films from zein and poly (lactic acid) for active food packaging. Food Hydrocoll..

[35] Busolo M.A., Torres-Giner S., Lagaron J.M. Enhancing the gas barrier properties of polylactic acid by means of electrospun ultrathin zein fibers. Proceedings of the 67th Annual Technical Conference.

[36] Lin D., Lu W., Kelly A.L., Zhang L., Zheng B., Miao S. (2017). Interactions of vegetable proteins with other polymers: Structure-function relationships and applications in the food industry. Trends Food Sci. Technol..

[37] Ganewatta M.S., Lokupitiya H.N., Tang C. (2019). Lignin Biopolymers in the Age of Controlled Polymerization. Polymers.

[38] Kun D., Pukánszky B. (2017). Polymer/lignin blends: Interactions, properties, applications. Eur. Polym. J..

[39] Li T., Takkellapati S. (2018). The current and emerging sources of technical lignins and their applications. Biofuels Bioprod. Biorefining.

[40] Huang J., Fu S., Gan L. (2019). Chapter 5-Lignin-Modified Thermoplastic Materials, Pages. Lignin Chemistry and Applications.

[41] Faruk O., Obaid N., Tjong J., Sain M., Faruk O., Sain M. (2016). 6-Lignin Reinforcement in Thermoplastic Composites. Lignin in Polymer Composites.

[42] Tanase C., Coşarcă S., Muntean D.L. (2019). A Critical Review of Phenolic Compounds Extracted from the Bark of Woody Vascular Plants and Their Potential Biological Activity. Molecules.

[43] Martinez-Abad A., Sanchez G., Ocio M.J., Lagaron J.M., Muñoz-Bonilla A., María L., Fernández-García M. (2013). Polymeric Materials Containing Natural Compounds with Antibacterial and Virucide Properties (Chapter 11). Polymeric Materials with Antimicrobial Activity: From Synthesis to Applications.

[44] Inamuddin (2016). Green Polymer Composites Technology: Properties and Applications.

[45] Valdés A., Mellinas A.C., Ramos M., Burgos N., Jiménez A., Garrigós M.D.C. (2015). Use of herbs, spices and their bioactive compounds in active food packaging. RSC Adv..

[46] García-Salinas S., Elizondo-Castillo H., Arruebo M., Mendoza G., Irusta S. (2018). Evaluation of the antimicrobial activity and cytotoxicity of different components of natural origin present in essential oils. Molecules.

[47] Palombo E.A. (2011). Traditional medicinal plant extracts and natural products with activity against oral bacteria: Potential application in the prevention and treatment of oral diseases. Evid.-Based Compl. Alt. Med..

[48] Vasile C., Sivertsvik M., Miteluţ A., Brebu M., Stoleru E., Rosnes J., Tanase E.E., Khan W., Pamfil D., Cornea P.C. (2017). Comparative analysis of the composition and active property evaluation of certain essential oils to assess their potential applications in active food packaging. Materials.

[49] Elieh-Ali-Komi D., Hamblin M.R. (2016). Chitin and chitosan: Production and application of versatile biomedical nanomaterials. Int. J. Adv. Res..

[50] Casadidio C., Peregrina D.V., Gigliobianco M.R., Deng S., Censi R., Di Martino P. (2019). Chitin and Chitosans: Characteristics, Eco-Friendly Processes, and Applications in Cosmetic Science. Mar. Drugs.

[51] Salaberria A.M., Labidi J., Fernandes S.C. (2014). Chitin nanocrystals and nanofibers as nano-sized fillers into thermoplastic starch-based biocomposites processed by melt-mixing. Chem. Eng. J..

[52] Coltelli M.B., Cinelli P., Gigante V., Aliotta L., Morganti P., Panariello L., Lazzeri A. (2019). Chitin Nanofibrils in Poly (Lactic Acid) (PLA) Nanocomposites: Dispersion and Thermo-Mechanical Properties. Int. J. Mol. Sci..

[53] Kawano A., Yamamoto K., Kadokawa J.I. (2017). Preparation of self-assembled chitin nanofiber-natural rubber composite sheets and porous materials. Biomolecules.

[54] Torres-Hernández Y., Ortega-Díaz G., Téllez-Jurado L., Castrejón-Jiménez N., Altamirano-Torres A., García-Pérez B., Balmori-Ramírez H. (2018). Biological compatibility of a polylactic acid composite reinforced with natural chitosan obtained from shrimp waste. Materials.

[55] Petchsoongsakul T., Dittanet P., Loykulnant S., Kongkaew C., Prapainainar P. (2019). Synthesis of Natural Composite of Natural Rubber Filling Chitosan Nanoparticles. Key Eng. Mater..

[56] de Barros-Alexandrino T.T., Tosi M.M., Assis O.B.G. (2019). Comparison Between Chitosan Nanoparticles and Cellulose Nanofibers as Reinforcement Fillers in Papaya Puree Films: Effects on Mechanical, Water Vapor Barrier, and Thermal Properties. Polym. Eng. Sci..

[57] van den Broek L.A., Knoop R.J., Kappen F.H., Boeriu C.G. (2015). Chitosan films and blends for packaging material. Carbohydr. Polym..

[58] Szymańska E., Winnicka K. (2015). Stability of chitosan—A challenge for pharmaceutical and biomedical applications. Mar. Drugs.

[59] Taurino R., Sciancalepore C., Collini L., Bondi M., Bondioli F. (2018). Functionalization of PVC by chitosan addition: Compound stability and tensile properties. Compos. Part. B Eng..

[60] Rihayat T., Riskina S., Syahputra W. (2019). Formulation of Polyurethane with Bentonite-Chitosan as Filler Applied to Carbon Steel as an Antibacterial and Environmentally Friendly Paint. Proceedings of the IOP Conference Series: Materials Science and Engineering 2019. International Conference on Science and Innovated Engineering (I-COSINE).

[61] Onwulata C.I., Thomas A.E., Cooke P.H. (2009). Effects of biomass in polyethylene or polylactic acid composites. J. Biobased Mater. Bioenergy.

[62] Schmidt M.M., Dornelles R.C.P., Mello R.O., Kubota E.H., Mazutti M.A., Kempka A.P., Demiate I.M. (2016). Collagen extraction process. Int. Food Res. J..

[63] Turnbull G., Clarke J., Picard F., Riches P., Jia L., Han F., Li B., Shu W. (2018). 3D bioactive composite scaffolds for bone tissue engineering. Bioact. Mater..

[64] Iqbal B., Muhammad N., Rahim A., Iqbal F., Sharif F., Safi S.Z., Khan A.S., Gonfa G., Uroos M., Rehman I.U. (2019). Development of collagen/PVA composites patches for osteochondral defects using a green processing of ionic liquid. Int. J. Polym. Mater. Polym. Biomater..

[65] Bergaya F., Lagaly G., Bergaya F., Lagaly G. (2013). Purification of natural clays (Chapter 7.1). Developments in Clay Science, Handbook of Clay Science.

[66] Guo F., Aryana S., Han Y., Jiao Y. (2018). A review of the synthesis and applications of polymer—Nanoclay composites. Appl. Sci..

[67] Shakeri F., Nodehi A., Atai M. (2019). PMMA/double-modified organoclay nanocomposites as fillers for denture base materials with improved mechanical properties. J. Mech. Behav. Biomed. Mater..

[68] Peña-Parás L., Sánchez J.A., Vidaltamayo R., Torres-Martínez L.M., Kharissova O.V., Kharissov B.I. (2019). Nanoclays for biomedical applications. Handbook of Ecomaterials.

[69] Berni R., Cai G., Hausman J.F., Guerriero G. (2019). Plant Fibers and Phenolics: A Review on Their Synthesis, Analysis and Combined Use for Biomaterials with New Properties. Fibers.

[70] Sharma A., Thakur M., Bhattacharya M., Mandal T., Goswami S. (2019). Commercial Application of Cellulose Nano-composites—A review. Biotechnol. Rep..

[71] Guerriero G., Berni R., Muñoz-Sanchez J., Apone F., Abdel-Salam E., Qahtan A., Alatar A.A., Cantini C., Cai G., Hausman J.F. (2018). Production of plant secondary metabolites: Examples, tips and suggestions for biotechnologists. Genes.

[72] Satyanarayana K.G., Arizaga G.G., Wypych F. (2009). Biodegradable composites based on lignocellulosic fibers—An overview. Prog. Polym. Sci..

[73] Ahmed B., Aboudi K., Tyagi V.K., Álvarez-Gallego C.J., Fernández-Güelfo L.A., Romero-García L.I., Kazmi A.A. (2019). Improvement of Anaerobic Digestion of Lignocellulosic Biomass by Hydrothermal Pretreatment. Appl. Sci..

[74] Pires J.R., de Souza V.G.L., Fernando A.L., Machado J., Soares F., Veiga G. (2019). Production of Nanocellulose from Lignocellulosic Biomass Wastes: Prospects and Limitations. Lecture Notes in Electrical Engineering, Innovation, Engineering and Entrepreneurship.

[75] Lee H.V., Hamid S.B.A., Zain S.K. (2014). Conversion of lignocellulosic biomass to nanocellulose: Structure and chemical process. Sci. World J..

[76] Yang J., Ching Y.C., Chuah C.H. (2019). Applications of Lignocellulosic Fibers and Lignin in Bioplastics: A Review. Polymers.

[77] Ching Y.C., Ershad A., Luqman C.A., Choo K.W., Yong C.K., Sabariah J.J., Chuah C.H., Liou N.S. (2016). Rheological properties of cellulose nanocrystal-embedded polymer composites: A review. Cellulose.

[78] Zarrinbakhsh N., Mohanty A.K., Misra M. (2019). Formulation optimization of bioreinforced composites from polyolefins and dried distillers’ grains using statistical methods. Compos. Part. A Appl. Sci. Manuf..

[79] Liu M., Thygesen A., Summerscales J., Meyer A.S. (2017). Targeted pre-treatment of hemp bast fibres for optimal performance in biocomposite materials: A review. Ind. Crops Prod..

[80] Saradava B.J., Kathwadia A.J., Gorviyala A.D., Joshi V.K. (2016). Mechanical characterization of hemp fiber reinforced polyester composites: A review. J. Polym. Compos..

[81] Catto A.L., Júnior M.A.D., Hansen B., Francisquetti E.L., Borsoi C. (2019). Characterization of polypropylene composites using yerba mate fibers as reinforcing filler. Compos. Part. B Eng..

[82] Zhang Q., Li Y., Cai H., Lin X., Yi W., Zhang J. (2019). Properties comparison of high density polyethylene composites filled with three kinds of shell fibers. Results Phys..

[83] Pappu A., Pickering K.L., Thakur V.K. (2019). Manufacturing and characterization of sustainable hybrid composites using sisal and hemp fibres as reinforcement of poly (lactic acid) via injection moulding. Ind. Crops Prod..

[84] Spiridon I., Leluk K., Resmerita A.M., Darie R.N. (2015). Evaluation of PLA–lignin bioplastics properties before and after accelerated weathering. Compos. Part. B Eng..

[85] Yong M., Zhang Y., Sun S., Liu W. (2019). Properties of polyvinyl chloride (PVC) ultrafiltration membrane improved by lignin: Hydrophilicity and antifouling. J. Membr. Sci..

[86] Dabral S., Turberg M., Wanninger A., Bolm C., Hernández J. (2017). Mechanochemical Lignin-Mediated Strecker Reaction. Molecules.

[87] Pregi E., Kun D., Vu V., Pukánszky B. (2019). Structure evolution in poly (ethylene-co-vinyl alcohol)/lignin blends: Effect of interactions and composition. Eur. Polym. J..

[88] Kabir A.S., Li H., Yuan H., Kuboki T., Xu C.C. (2019). Effects of de-polymerized lignin content on thermo-oxidative and thermal stability of polyethylene. J. Anal. Appl. Pyrolysis.

[89] Nair S.S., Chen H., Peng Y., Huang Y., Yan N. (2018). Polylactic acid biocomposites reinforced with nanocellulose fibrils with high lignin content for improved mechanical, thermal, and barrier properties. ACS Sustain. Chem. Eng..

[90] Aqlil M., Moussemba Nzenguet A., Essamlali Y., Snik A., Larzek M., Zahouily M. (2017). Graphene oxide filled lignin/starch polymer bionanocomposite: Structural, physical, and mechanical studies. J. Agric. Food Chem..

[91] Souza de Miranda C., Ferreira M.S., Magalhães M.T., Gonçalves A.P.B., Carneiro de Oliveira J., Guimarães D.H., José N.M. (2015). Effect of the glycerol and lignin extracted from Piassava fiber in cassava and corn starch films. Mater. Res..

[92] Zadeh E.M., O’Keefe S.F., Kim Y.T. (2018). Utilization of lignin in biopolymeric packaging films. ACS Omega.

[93] Polat Y., Stojanovska E., Negawo T.A., Doner E., Kilic A., Jawaid M., Sapuan S., Alothman O. (2017). Lignin as an Additive for Advanced Composites. Green Biocomposites. Green Energy and Technology.

[94] Domínguez-Robles J., Martin N.K., Fong M.L., Stewart S.A., Irwin N.J., Rial-Hermida M.I., Donnelly R.F., Larrañeta E. (2019). Antioxidant PLA Composites Containing Lignin for 3D Printing Applications: A Potential Material for Healthcare Applications. Pharmaceutics.

[95] Nasir M., Hashim R., Sulaiman O., Asim M., Jawaid M., Boufi S., Abdul Khalil H.P.S. (2017). Nanocellulose: Preparation methods and applications. Cellulose-Reinforced Nanofibre Composites.

[96] Panchal P., Ogunsona E., Mekonnen T. (2019). Trends in advanced functional material applications of nanocellulose. Processes.

[97] Jamróz E., Kulawik P., Kopel P. (2019). The Effect of Nanofillers on the Functional Properties of Biopolymer-Based Films: A Review. Polymers.

[98] Vijay Kumar Thakur (2014). Nanocellulose Polymer Nanocomposites: Fundamentals and Applications.

[99] Yousri O.M., Abdellatif M.H., Bassioni G. (2018). Effect of Al_2_O_3_ Nanoparticles on the Mechanical and Physical Properties of Epoxy Composite. Arab. J. Sci. Eng..

[100] Karatrantos A., Clarke N., Kröger M. (2016). Modeling of polymer structure and conformations in polymer nanocomposites from atomistic to mesoscale: A Review. Polym. Rev..

[101] Bayer I.S., Fragouli D., Attanasio A., Sorce B., Bertoni G., Brescia R., Pompa P.P. (2011). Water-repellent cellulose fiber networks with multifunctional properties. ACS Appl. Mater. Interfaces.

[102] Hirn U., Schennach R. (2015). Comprehensive analysis of individual pulp fiber bonds quantifies the mechanisms of fiber bonding in paper. Sci. Rep..

[103] Mishra P.K., Ekielski A. (2019). The self-assembly of lignin and its application in nanoparticle synthesis: A short review. Nanomaterials.

[104] Yu H.Y., Zhang D.Z., Lu F.F., Yao J. (2016). New approach for single-step extraction of carboxylated cellulose nanocrystals for their use as adsorbents and flocculants. ACS Sustain. Chem. Eng..

[105] Shak K.P.Y., Pang Y.L., Mah S.K. (2018). Nanocellulose: Recent advances and its prospects in environmental remediation. Beilstein J. Nanotechnol..

[106] Ferreira F.V., Pinheiro I.F., de Souza S.F., Mei L.H., Lona L.M. (2019). Polymer Composites Reinforced with Natural Fibers and Nanocellulose in the Automotive Industry: A Short Review. J. Compos. Sci..

[107] Moberg T., Sahlin K., Yao K., Geng S., Westman G., Zhou Q., Rigdahl M. (2017). Rheological properties of nanocellulose suspensions: Effects of fibril/particle dimensions and surface characteristics. Cellulose.

[108] Tayeb A.H., Amini E., Ghasemi S., Tajvidi M. (2018). Cellulose nanomaterials—Binding properties and applications: A review. Molecules.

[109] Mishra R.K., Ha S.K., Verma K., Tiwari S.K. (2018). Recent progress in selected bio-nanomaterials and their engineering applications: An overview. J. Sci. Adv. Mater. Devices.

[110] Alila S., Besbes I., Vilar M.R., Mutjé P., Boufi S. (2013). Non-woody plants as raw materials for production of microfibrillated cellulose (MFC): A comparative study. Ind. Crops Prod..

[111] Bacakova L., Pajorova J., Bacakova M., Skogberg A., Kallio P., Kolarova K., Svorcik V. (2019). Versatile Application of Nanocellulose: From Industry to Skin Tissue Engineering and Wound Healing. Nanomaterials.

[112] Wulandari W.T., Rochliadi A., Arcana I.M. (2016). Nanocellulose prepared by acid hydrolysis of isolated cellulose from sugarcane bagasse. Proceedings of the IOP conference series: Materials science and engineering 2016. 10th Joint Conference on Chemistry.

[113] Mokhena T., Sefadi J., Sadiku E., John M., Mochane M., Mtibe A. (2018). Thermoplastic processing of PLA/cellulose nanomaterials composites. Polymers.

[114] Chen Y., Wu Q., Ai X., Huang M., Lu Q. (2017). Sono-chemical preparation of cellulose nanowhiskers from Luffa Cylindrica fibers optimized by response surface methodology. Cell. Chem. Technol..

[115] Santos A.S., Pereira-da-Silva M.A., Oliveira J.E., Mattoso L.H., Medeiros E.S. (2016). Accelerated sonochemical extraction of cellulose nanowhiskers. J. Nanosci. Nanotechnol..

[116] Ribeiro R.S., Pohlmann B.C., Calado V., Bojorge N., Pereira N. (2019). Production of nanocellulose by enzymatic hydrolysis: Trends and challenges. Eng. Life Sci..

[117] El Achaby M., Kassab Z., Aboulkas A., Gaillard C., Barakat A. (2018). Reuse of red algae waste for the production of cellulose nanocrystals and its application in polymer nanocomposites. Int. J. Biol. Macromol..

[118] Kawee N., Lam N.T., Sukyai P. (2018). Homogenous isolation of individualized bacterial nanofibrillated cellulose by high pressure homogenization. Carbohyd. Polym..

[119] Davoudpour Y., Hossain S., Khalil H.A., Haafiz M.M., Ishak Z.M., Hassan A., Sarker Z.I. (2015). Optimization of high pressure homogenization parameters for the isolation of cellulosic nanofibers using response surface methodology. Ind. Crops Prod..

[120] Jacob J., Peter G., Thomas S., Haponiuk J.T., Gopi S. (2019). Chitosan and polyvinyl alcohol nanocomposites with cellulose nanofibers from ginger rhizomes and its antimicrobial activities. Int. J. Biol. Macromol..

[121] Oh J.W., Chun S., Chandrasekaran M. (2019). Preparation and In Vitro Characterization of Chitosan Nanoparticles and Their Broad-Spectrum Antifungal Action Compared to Antibacterial Activities against Phytopathogens of Tomato. Agronomy.

[122] de Araújo Braz E.M., e Silva S.C.C.C., da Silva D.A., de Amorim Carvalho F.A., Barreto H.M., Júnior L.D.S.S., da Silva Filho E.C. (2018). Modified chitosan-based bioactive material for antimicrobial application: Synthesis and characterization. Int. J. Biol. Macromol..

[123] Wang Z., Yao Z., Zhou J., He M., Jiang Q., Li A., Zhang D. (2019). Improvement of polylactic acid film properties through the addition of cellulose nanocrystals isolated from waste cotton cloth. Int. J. Biol. Macromol..

[124] Tekinalp H.L., Meng X., Lu Y., Kunc V., Love L.J., Peter W.H., Ozcan S. (2019). High modulus biocomposites via additive manufacturing: Cellulose nanofibril networks as “microsponges”. Compos. Part. B Eng..

[125] Voisin H., Bergström L., Liu P., Mathew A. (2017). Nanocellulose-based materials for water purification. Nanomaterials.

[126] Curvello R., Raghuwanshi V.S., Garnier G. (2019). Engineering nanocellulose hydrogels for biomedical applications. Adv. Coll. Interface Sci..

[127] Mishra R.K., Sabu A., Tiwari S.K. (2018). Materials chemistry and the futurist eco-friendly applications of nanocellulose: Status and prospect. J. Saudi Chem. Soc..

[128] Chakrabarty A., Teramoto Y. (2018). Recent advances in nanocellulose composites with polymers: A guide for choosing partners and How to incorporate them. Polymers.

[129] Alghamdi H., Nair S.A., Neithalath N. (2019). Insights into material design, extrusion rheology, and properties of 3D-printable alkali-activated fly ash-based binders. Mater. Design.

[130] de Oliveira A.D., Beatrice C.A.G., Sivasankaran S. (2019). Polymer Nanocomposites with Different Types of Nanofiller. Nanocomposites-Recent Evolutions.

[131] Pelin C.E., Stefan A., Pelin G., Dinca I., Ficai A., Andronescu E., Voicu G. (2015). Mechanical Properties of Nanofilled Polypropylene Composites. INCAS Bull..

[132] Irvin C.W., Satam C.C., Meredith J.C., Shofner M.L. (2019). Mechanical reinforcement and thermal properties of PVA tricomponent nanocomposites with chitin nanofibers and cellulose nanocrystals. Compos. Part. A Appl. Sci. Manuf..

[133] Mok C.F., Ching Y.C., Muhamad F., Abu Osman N.A., Singh R. (2017). Poly (vinyl alcohol)-α-chitin composites reinforced by oil palm empty fruit bunch fiber-derived nanocellulose. Int. J. Polym. Anal. Charact..

[134] Echave M.C., Pimenta-Lopes C., Pedraz J.L., Mehrali M., Dolatshahi-Pirouz A., Ventura F., Orive G. (2019). Enzymatic Crosslinked Gelatin 3D Scaffolds for Bone Tissue Engineering. Int. J. Pharm..

[135] Yang G., Xiao Z., Long H., Ma K., Zhang J., Ren X., Zhang J. (2018). Assessment of the characteristics and biocompatibility of gelatin sponge scaffolds prepared by various crosslinking methods. Sci. Rep..

[136] Roseti L., Parisi V., Petretta M., Cavallo C., Desando G., Bartolotti I., Grigolo B. (2017). Scaffolds for bone tissue engineering: State of the art and new perspectives. Mater. Sci. Eng. C.

[137] Zhao Y., Huerta R.R., Saldaña M.D. (2019). Use of subcritical water technology to develop cassava starch/chitosan/gallic acid bioactive films reinforced with cellulose nanofibers from canola straw. J. Supercrit. Fluids.

[138] Lin D., Kuang Y., Chen G., Kuang Q., Wang C., Zhu P., Fang Z. (2017). Enhancing moisture resistance of starch-coated paper by improving the film forming capability of starch film. Ind. Crops Prod..

[139] Zhao Y., Saldaña M.D. (2019). Hydrolysis of cassava starch, chitosan and their mixtures in pressurized hot water media. J. Supercrit. Fluids.

[140] Prochoń M., Marzec A., Szadkowski B. (2019). Preparation and Characterization of New Environmentally Friendly Starch-Cellulose Materials Modified with Casein or Gelatin for Agricultural Applications. Materials.

[141] Yang J., Kwon G.J., Hwang K., Kim D.Y. (2018). Cellulose–Chitosan Antibacterial Composite Films Prepared from LiBr Solution. Polymers.

[142] Hernández-López M., Correa-Pacheco Z.N., Bautista-Baños S., Zavaleta-Avejar L., Benítez-Jiménez J.J., Sabino-Gutiérrez M.A., Ortega-Gudiño P. (2019). Bio-based composite fibers from pine essential oil and PLA/PBAT polymer blend. Morphological, physicochemical, thermal and mechanical characterization. Mater. Chem. Phys..

[143] Sango T., Stoclet G., Joly N., Marin A., Yona A.M.C., Duchatel L., Ndikontar K.M., Lefebvre J.M. (2019). Water–soluble extracts from banana pseudo–stem as functional additives for polylactic acid: Thermal and mechanical investigations. Eur. Polym. J..

[144] Kostic D., Vukasinovic-Sekulic M., Armentano I., Torre L., Obradovic B. (2019). Multifunctional ternary composite films based on PLA and Ag/alginate microbeads: Physical characterization and silver release kinetics. Mater. Sci. Eng. C.

[145] Ravichandran S., Radhakrishnan J., Jayabal P., Venkatasubbu G.D. (2019). Antibacterial screening studies of electrospun Polycaprolactone nano fibrous mat containing Clerodendrum phlomidis leaves extract. Appl. Surf. Sci..

[146] Abdelghany A.M., Oraby A.H., Asnag G.M. (2019). Structural, thermal and electrical studies of polyethylene oxide/starch blend containing green synthesized gold nanoparticles. J. Mol. Struct..

[147] Greco A., Ferrari F., Maffezzoli A. (2019). Mechanical properties of poly (lactid acid) plasticized by cardanol derivatives. Polym. Degrad. Stab..

[148] Gama N., Ferreira A., Barros-Timmons A. (2019). 3D printed cork/polyurethane composite foams. Mater. Des..

[149] Lizárraga-Laborín L.L., Quiroz-Castillo J.M., Encinas-Encinas J.C., Castillo-Ortega M.M., Burruel-Ibarra S.E., Romero-García J., Torres-Ochoa J.A., Cabrera-Germán D., Rodríguez-Félix D.E. (2018). Accelerated weathering study of extruded polyethylene/poly (lactic acid)/chitosan films. Polym. Degrad. Stab..

[150] Mahajan P.V., Caleb O.J., Singh Z., Watkins C.B., Geyer M. (2014). Postharvest treatments of fresh produce. Philos. Trans. R. Soc. A Math. Phy. Eng. Sci..

[151] Sandarani M.D.J.C., Dasanayaka D.C.M.C.K., Jayasinghe C.V.L. (2018). Strategies Used to Prolong the Shelf Life of Fresh Commodities. J. Agric. Sci. Food Res..

[152] Zambrano-Zaragoza M.L., González-Reza R., Mendoza-Muñoz N., Miranda-Linares V., Bernal-Couoh T.F., Mendoza-Elvira S., Quintanar-Guerrero D. (2018). Nanosystems in edible coatings: A novel strategy for food preservation. Int. J. Mol. Sci..

[153] Yildirim S., Röcker B., Pettersen M.K., Nilsen-Nygaard J., Ayhan Z., Rutkaite R., Radusin T., Suminska P., Marcos B., Coma V. (2018). Active packaging applications for food. Compr. Rev. Food Sci. Food Saf..

[154] Valdés A., Mellinas A.C., Ramos M., Garrigós M.C., Jiménez A. (2014). Natural additives and agricultural wastes in biopolymer formulations for food packaging. Front. Chem..

[155] Soto M.L., Moure A., Domínguez H., Parajó J.C. (2011). Recovery, concentration and purification of phenolic compounds by adsorption: A review. J. Food Eng..

[156] Guo Y., Chen X., Yang F., Wang T., Ni M., Chen Y., Yang F., Huang D., Fu C., Wang S. (2019). Preparation and Characterization of Chitosan-Based Ternary Blend Edible Films with Efficient Antimicrobial Activities for Food Packaging Applications. J. Food Sci..

[157] Yun D., Cai H., Liu Y., Xiao L., Song J., Liu J. (2019). Development of active and intelligent films based on cassava starch and Chinese bayberry (Myrica rubra Sieb. et Zucc.) anthocyanins. RSC Adv..

[158] de Oliveira Filho J.G., Rodrigues J.M., Valadares A.C.F., de Almeida A.B., de Lima T.M., Takeuchi K.P., Alves C.C.F., de Figueiredo Sousa H.A., da Silva E.R., Dyszy F.B. (2019). Active food packaging: Alginate films with cottonseed protein hydrolysates. Food Hydrocoll..

[159] Munteanu B.S., Dumitriu R.P., Profire L., Sacarescu L., Hitruc G.E., Stoleru E., Dobromir M., Matricala A.L., Vasile C. (2016). Hybrid nanostructures containing sulfadiazine modified chitosan as antimicrobial drug carriers. Nanomaterials.

[160] Munteanu B.S., Aytac Z., Pricope G.M., Uyar T., Vasile C. (2014). Polylactic acid (PLA)/Silve r-NP/VitaminE bionanocomposite electrospun nanofibers with antibacterial and antioxidant activity. J. Nanopart. Res..

[161] Macocinschi D., Filip D., Paslaru E., Munteanu B.S., Dumitriu R.P., Pricope G.M., Aflori M., Dobromir M., Nica V., Vasile C. (2015). Polyurethane–extracellular matrix/silver bionanocomposites for urinary catheters. J. Bioact. Compat. Pol..

[162] Filip D., Macocinschi D., Paslaru E., Munteanu B.S., Dumitriu R.P., Lungu M., Vasile C. (2014). Polyurethane biocompatible silver bionanocomposites for biomedical applications. J. Nanopart. Res..

[163] Munteanu B.S., Sacarescu L., Vasiliu A.L., Hitruc G.E., Pricope G.M., Sivertsvik M., Rosnes J.T., Vasile C. (2018). Antioxidant/antibacterial electrospun nanocoatings applied onto PLA films. Materials.

[164] Vasile C., Darie R.N., Sdrobis A., Pâslaru E., Pricope G., Baklavaridis A., Munteanu B.S., Zuburtikudis I. (2014). Effectiveness of chitosan as antimicrobial agent in LDPE/CS composite films as minced poultry meat packaging materials. Cell. Chem. Technol..

[165] Munteanu B.S., Paslaru E., Zemljic L.F., Sdrobis A., Pricope G.M., Vasile C. (2014). Chitosan coatings applied to polyethylene surface to obtain food-packaging materials. Cell. Chem. Technol..

[166] Jeevahan J., Chandrasekaran M., Durairaj R.B., Mageshwaran G., Joseph G.B. (2017). A Brief Review on Edible Food Packing Materials. J. Global Eng. Probl. Solut..

[167] Hassan B., Chatha S.A.S., Hussain A.I., Zia K.M., Akhtar N. (2018). Recent advances on polysaccharides, lipids and protein based edible films and coatings: A review. Int. J. Biol. Macromol..

[168] Villar M.A., Barbosa S.E., Alejandra García M.A., Castillo L.A., López O.V. (2017). Starch-Based Materials in Food Packaging: Processing, Characterization and Applications.

[169] Yusof N.M., Jai J., Hamzah F. (2019). Effect of Coating Materials on the Properties of Chitosan-Starch-Based Edible Coatings. Proceedings of the IOP Conference Series: Materials science and engineering, 5th International Conference on Advanced Engineering and Technology (5th ICAET).

[170] Escamilla-García M., Reyes-Basurto A., García-Almendárez B., Hernández-Hernández E., Calderón-Domínguez G., Rossi-Márquez G., Regalado-González C. (2017). Modified starch-chitosan edible films: Physicochemical and mechanical characterization. Coatings.

[171] Parreidt T., Müller K., Schmid M. (2018). Alginate-based edible films and coatings for food packaging applications. Foods.

[172] Wang H., Qian J., Ding F. (2018). Emerging chitosan-based films for food packaging applications. J. Agric. Food Chem..

[173] Chan E.S. (2011). Preparation of Ca-alginate beads containing high oil content: Influence of process variables on encapsulation efficiency and bead properties. Carbohydr. Polym..

[174] Chan E.S., Wong S.L., Lee P.P., Lee J.S., Ti T.B., Zhang Z., Poncelet D., Ravindra P., Phan S.H., Yim Z.H. (2011). Effects of starch filler on the physical properties of lyophilized calcium–alginate beads and the viability of encapsulated cells. Carbohydr. Polym..

[175] Fujiwara G.M., Campos R., Costa C.K., Dias J.F.G., Miguel O.G., Miguel M.D., Marques F.A., Zanin S.M.W. (2013). Production and characterization of alginate-starch-chitosan microparticles containing stigmasterol through the external ionic gelation technique. Braz. J. Pharm. Sci..

[176] Homayouni A., Azizi A., Ehsani M.R., Yarmand M.S., Razavi S.H. (2008). Effect of microencapsulation and resistant starch on the probiotic survival and sensory properties of synbiotic ice cream. Food Chem..

[177] Xie Y.L., Jiang W., Li F., Zhang Y., Liang X.Y., Wang M., Zhou X., Wu S.Y., Zhang C.H. (2019). Controlled Release of Spirotetramat Using Starch–Chitosan–Alginate-Encapsulation. Bull. Environ. Contam. Toxicol..

[178] de Araújo Etchepare M., Raddatz G.C., de Moraes Flores E.M., Zepka L.Q., Jacob-Lopes E., Barin J.S., Ferreira Grosso C.R., de Menezes C.R. (2016). Effect of resistant starch and chitosan on survival of Lactobacillus acidophilus microencapsulated with sodium alginate. LWT-Food Sci. Technol..

[179] Nnamonu L.A., Sha’Ato R., Onyido I. (2012). Alginate Reinforced Chitosan and Starch Beads in Slow Release Formulation of Imazaquin Herbicide—Preparation and Characterization. Mater. Sci. Appl..

[180] Kumar P., Sethi S., Sharma R.R., Singh S., Varghese E. (2018). Improving the shelf life of fresh-cut ‘Royal Delicious’ apple with edible coatings and anti-browning agents. J. Food Sci. Technol..

[181] Valdés A., Ramos M., Beltrán A., Jiménez A., Garrigós M. (2017). State of the art of antimicrobial edible coatings for food packaging applications. Coatings.

[182] Liu J., Meng C.G., Liu S., Kan J., Jin C.H. (2017). Preparation and characterization of protocatechuic acid grafted chitosan films with antioxidant activity. Food Hydrocoll..

[183] Ruiz-Cruz S., Valenzuela-Lopez C.C., Chaparro-Hernandez S., Ornelas-Paz J.D.J., Toro-Sanchez C.L.D., Marquez-Rios E., Lopez-Mata M.A., Odcanod-Hioguera V.M., Valdez-Hurtado S. (2019). Effects of chitosan-tomato plant extract edible coatings on the quality and shelf life of chicken fillets during refrigerated storage. Food Sci. Technol..

[184] Ramírez-Guerra H.E., Castillo-Yañez F.J., Montaño-Cota E.A., Ruíz-Cruz S., Márquez-Ríos E., Canizales-Rodríguez D.F., Torres-Arreola W., Montoya-Camacho N., Ocaño-Higuera V.M. (2018). Protective Effect of an Edible Tomato Plant Extract/Chitosan Coating on the Quality and Shelf Life of Sierra Fish Fillets. J. Chem..

[185] Kaya M., Khadem S., Cakmak Y.S., Mujtaba M., Ilk S., Akyuz L., Salaberria A.M., Labidi J., Abdulqadir A.H., Deligöz E. (2018). Antioxidative and antimicrobial edible chitosan films blended with stem, leaf and seed extracts of Pistacia terebinthus for active food packaging. RSC Adv..

[186] Chen C., Nie Z., Wan C., Chen J. (2019). Preservation of Xinyu tangerines with an edible coating using Ficus hirta Vahl. fruits extract-incorporated chitosan. Biomolecules.

[187] Karami N., Kamkar A., Sahbazi Y., Misaghi A. (2019). Edible films based on chitosan-flaxseed mucilage: In vitro antimicrobial and antioxidant properties and their application on survival of food-borne pathogenic bacteria in raw minced trout fillets. Pharm. Biomed. Res..

[188] Azimzadeh B., Jahadi M. (2018). Effect of chitosan edible coating with Laurus nobilis extract on shelf life of cashew. Food Sci. Nutr..

[189] Chen C., Peng X., Zeng R., Chen M., Wan C., Chen J. (2016). Ficus hirta fruits extract incorporated into an alginate-based edible coating for Nanfeng mandarin preservation. Sci. Hortic..

[190] Nair M.S., Saxena A., Kaur C. (2018). Effect of chitosan and alginate based coatings enriched with pomegranate peel extract to extend the postharvest quality of guava (Psidium guajava L.). Food Chem..

[191] Yang G., Yue J., Gong X., Qian B., Wang H., Deng Y., Zhao Y. (2014). Blueberry leaf extracts incorporated chitosan coatings for preserving postharvest quality of fresh blueberries. Postharvest Biol. Technol..

[192] Siracusa V., Romani S., Gigli M., Mannozzi C., Cecchini J., Tylewicz U., Lotti N. (2018). Characterization of Active Edible Films based on Citral Essential Oil, Alginate and Pectin. Materials.

[193] Tesfay S.Z., Magwaza L.S. (2017). Evaluating the efficacy of moringa leaf extract, chitosan and carboxymethyl cellulose as edible coatings for enhancing quality and extending postharvest life of avocado (Persea americana Mill.) fruit. Food Packag. Shelf Life.

[194] Duran M., Aday M.S., Zorba N.N.D., Temizkan R., Büyükcan M.B., Caner C. (2016). Potential of antimicrobial active packaging ‘containing natamycin, nisin, pomegranate and grape seed extract in chitosan coating’to extend shelf life of fresh strawberry. Food Bioprod. Process..

[195] Castro-López C., Sánchez-Alejo E.J., Saucedo-Pompa S., Rojas R., Aranda-Ruiz J., Martínez-Avila G.C.G. (2016). Fluctuations in phenolic content, ascorbic acid and total carotenoids and antioxidant activity of fruit beverages during storage. Heliyon.

[196] Baltacioglu C., Velioglu S., Karacabey E. (2011). Changes in total phenolic and flavonoid contents of rowanberry fruit during postharvest storage. J. Food Qual..

[197] Díaz-Mula H.M., Serrano M., Valero D. (2012). Alginate coatings preserve fruit quality and bioactive compounds during storage of sweet cherry fruit. Food Bioprocess. Technol..

[198] Cofelice M., Lopez F., Cuomo F. (2019). Quality Control of Fresh-Cut Apples after Coating Application. Foods.

[199] Peretto G., Du W.X., Avena-Bustillos R.J., Berrios J.D.J., Sambo P., McHugh T.H. (2017). Electrostatic and conventional spraying of alginate-based edible coating with natural antimicrobials for preserving fresh strawberry quality. Food Bioprocess. Technol..

[200] Davoodi F., Naji M.H. (2018). Study of the effect of sodium alginate coating containing pomegranate peel extract on chemical, sensory and microbial quality of walnut kernel. Environ. Health Eng. Manag. J..

[201] Pagliarulo C., Sansone F., Moccia S., Russo G.L., Aquino R.P., Salvatore P., Di Stasio M., Volpe M.G. (2016). Preservation of strawberries with an antifungal edible coating using peony extracts in chitosan. Food Bioprocess. Technol..

[202] Akram N.A., Shafiq F., Ashraf M. (2017). Ascorbic acid-a potential oxidant scavenger and its role in plant development and abiotic stress tolerance. Front. Plant. Sci..

[203] Nie Z., Wan C., Chen C., Chen J. (2019). Comprehensive Evaluation of the Postharvest Antioxidant Capacity of Majiayou Pomelo Harvested at Different Maturities Based on PCA. Antioxidants.

[204] Peralta J., Bitencourt-Cervi C.M., Maciel V.B., Yoshida C.M., Carvalho R.A. (2019). Aqueous hibiscus extract as a potential natural pH indicator incorporated in natural polymeric films. Food Packag. Shelf Life.

[205] Wang X., Yong H., Gao L., Li L., Jin M., Liu J. (2019). Preparation and characterization of antioxidant and pH-sensitive films based on chitosan and black soybean seed coat extract. Food Hydrocoll..

[206] Panche A.N., Diwan A.D., Chandra S.R. (2016). Flavonoids: An overview. J. Nutr. Sci..

[207] Müller P., Schmid M. (2019). Intelligent packaging in the food sector: A brief overview. Foods.

[208] Zhai X., Li Z., Zhang J., Shi J., Zou X., Huang X., Zhang D., Sun Y., Yang Z., Holmes M. (2018). Natural biomaterial-based edible and pH-sensitive films combined with electrochemical writing for intelligent food packaging. J. Agric. Food Chem..

[209] Halász K., Csóka L. (2018). Black chokeberry (Aronia melanocarpa) pomace extract immobilized in chitosan for colorimetric pH indicator film application. Food Packag. Shelf Life.

[210] Frond A.D., Iuhas C.I., Stirbu I., Leopold L., Socaci S., Stănilă A., Ayvaz H., Socaciu A., Socaciu M., Diaconeasa Z. (2019). Phytochemical Characterization of Five Edible Purple-Reddish Vegetables: Anthocyanins, Flavonoids, and Phenolic Acid Derivatives. Molecules.

[211] Martín J., Kuskoski E.M., Navas M.J., Asuero A.G., Justiono J. (2017). Antioxidant capacity of anthocyanin pigments. Flavonoids: From Biosynthesis to Human Health.

[212] Khoo H.E., Azlan A., Tang S.T., Lim S.M. (2017). Anthocyanidins and anthocyanins: Colored pigments as food, pharmaceutical ingredients, and the potential health benefits. Food Nutr. Res..

[213] Yousuf B., Gul K., Wani A.A., Singh P. (2016). Health benefits of anthocyanins and their encapsulation for potential use in food systems: A review. Crit. Rev. Food Sci. Nutr..

[214] Li S., Wu B., Fu W., Reddivari L. (2019). The Anti-inflammatory Effects of Dietary Anthocyanins against Ulcerative Colitis. Int. J. Mol. Sci..

[215] Khoo H.E., Lim S.M., Azlan A. (2019). Evidence-Based Therapeutic Effects of Anthocyanins from Foods. Pak. J. Nutr..

[216] Xiu-li H.E., Xue-li L.I., Yuan-ping L.V., Qiang H.E. (2015). Composition and color stability of anthocyanin-based extract from purple sweet potato. Food Sci. Technol. (Campinas).

[217] Silva S., Costa E.M., Calhau C., Morais R.M., Pintado M.E. (2017). Anthocyanin extraction from plant tissues: A review. Crit. Rev. Food Sci. Nutr..

[218] Ma Q., Liang T., Cao L., Wang L. (2018). Intelligent poly (vinyl alcohol)-chitosan nanoparticles-mulberry extracts films capable of monitoring pH variations. Int. J. Biol. Macromol..

[219] Jiang T., Mao Y., Sui L., Yang N., Li S., Zhu Z., Wang C., Yin S., He J., He Y. (2019). Degradation of anthocyanins and polymeric color formation during heat treatment of purple sweet potato extract at different pH. Food Chem..

[220] Yong H., Wang X., Bai R., Miao Z., Zhang X., Liu J. (2019). Development of antioxidant and intelligent pH-sensing packaging films by incorporating purple-fleshed sweet potato extract into chitosan matrix. Food Hydrocoll..

[221] Vo T.V., Dang T.H., Chen B.H. (2019). Synthesis of Intelligent pH Indicative Films from Chitosan/Poly (vinyl alcohol)/Anthocyanin Extracted from Red Cabbage. Polymers.

[222] He B., Ge J., Yue P., Yue X., Fu R., Liang J., Gao X. (2017). Loading of anthocyanins on chitosan nanoparticles influences anthocyanin degradation in gastrointestinal fluids and stability in a beverage. Food Chem..

[223] Shafiee-Jood M., Cai X. (2016). Reducing food loss and waste to enhance food security and environmental sustainability. Environ. Sci. Technol..

[224] Lobo V., Patil A., Phatak A., Chandra N. (2010). Free radicals, antioxidants and functional foods: Impact on human health. Pharmacogn. Rev..

[225] Vital A.C.P., Guerrero A., Kempinski E.M.B.C., de Oliveira Monteschio J., Sary C., Ramos T.R., Campo M.D.M., do Prado I.N. (2018). Consumer profile and acceptability of cooked beef steaks with edible and active coating containing oregano and rosemary essential oils. Meat Sci..

[226] Yahaya W., Amnin W., Abu Yazid N., Azman M., Aini N., Almajano M.P. (2019). Antioxidant Activities and Total Phenolic Content of Malaysian Herbs as Components of Active Packaging Film in Beef Patties. Antioxidants.

[227] Difonzo G., Squeo G., Calasso M., Pasqualone A., Caponio F. (2019). Physico-Chemical, Microbiological and Sensory Evaluation of Ready-to-Use Vegetable Pâté Added with Olive Leaf Extract. Foods.

[228] Kaurinovic B., Vastag D., Shalaby E. (2019). Flavonoids and Phenolic Acids as Potential Natural Antioxidants. Antioxidants.

[229] Santos-Sánchez N.F., Salas-Coronado R., Villanueva-Cañongo C., Hernández-Carlos B., Shalaby E. (2019). Antioxidant Compounds and Their Antioxidant Mechanism. Antioxidants.

[230] Kähkönen M.P., Hopia A.I., Vuorela H.J., Rauha J.P., Pihlaja K., Kujala T.S., Heinonen M. (1999). Antioxidant activity of plant extracts containing phenolic compounds. J. Agric. Food Chem..

[231] Hammer K.A., Carson C.F., Riley T.V. (1999). Antimicrobial activity of essential oils and other plant extracts. J. Appl. Microbiol..

[232] Xu D.P., Li Y., Meng X., Zhou T., Zhou Y., Zheng J., Zhang J.J., Li H.-B. (2017). Natural antioxidants in foods and medicinal plants: Extraction, assessment and resources. Int. J. Mol. Sci..

[233] Pabón-Baquero L.C., Otálvaro-Álvarez Á.M., Fernández M.R.R., Chaparro-González M.P., Shalaby E. (2019). Plant Extracts as Antioxidant Additives for Food Industry. Antioxidants in Foods and Its Applications.

[234] Souza V.G.L., Rodrigues P.F., Duarte M.P., Fernando A.L. (2018). Antioxidant migration studies in chitosan films incorporated with plant extracts. J. Renew. Mater..

[235] Benbettaïeb N., Debeaufort F., Karbowiak T. (2019). Bioactive edible films for food applications: Mechanisms of antimicrobial and antioxidant activity. Crit. Rev. Food Sci. Nutr..

[236] Benbettaïeb N., Chambin O., Assifaoui A., Al-Assaf S., Karbowiak T., Debeaufort F. (2016). Release of coumarin incorporated into chitosan-gelatin irradiated films. Food Hydrocoll..

[237] Ulewicz-Magulska B., Wesolowski M. (2019). Total phenolic contents and antioxidant potential of herbs used for medical and culinary purposes. Plant. Foods Hum. Nutr..

[238] El-Sayed S.M., Youssef A.M. (2019). Potential application of herbs and spices and their effects in functional dairy products. Heliyon.

[239] Jiang T.A. (2019). Health Benefits of Culinary Herbs and Spices. J. AOAC Int..

[240] Proestos C., Varzakas T. (2017). Aromatic plants: Antioxidant capacity and polyphenol characterisation. Foods.

[241] Ghandahari Yazdi A.P., Barzegar M., Sahari M.A., Ahmadi Gavlighi H. (2019). Optimization of the enzyme-assisted aqueous extraction of phenolic compounds from pistachio green hull. Food Sci. Nutr..

[242] Tchabo W., Ma Y., Kwaw E., Xiao L., Wu M., Apaliya M. (2018). Impact of extraction parameters and their optimization on the nutraceuticals and antioxidant properties of aqueous extract mulberry leaf. Int. J. Food Prop..

[243] Perazzo K.K.N.C.L., de Vasconcelos Conceição A.C., dos Santos J.C.P., de Jesus Assis D., Souza C.O., Druzian J.I. (2014). Properties and antioxidant action of actives cassava starch films incorporated with green tea and palm oil extracts. PLoS ONE.

[244] Talón E., Trifkovic K.T., Nedovic V.A., Bugarski B.M., Vargas M., Chiralt A., González-Martínez C. (2017). Antioxidant edible films based on chitosan and starch containing polyphenols from thyme extracts. Carbohyd. Polym..

[245] Chawda P.J., Shi J., Xue S., Young Quek S. (2017). Co-encapsulation of bioactives for food applications. Food Qual. Safety.

[246] Kanokpanont S., Yamdech R., Aramwit P. (2018). Stability enhancement of mulberry-extracted anthocyanin using alginate/chitosan microencapsulation for food supplement application. Artif. Cell. Nanomedicine Biotechnol..

[247] Aloui H., Khwaldia K., Sánchez-González L., Muneret L., Jeandel C., Hamdi M., Desobry S. (2014). Alginate coatings containing grapefruit essential oil or grapefruit seed extract for grapes preservation. Int. J. Food Sci. Technol..

[248] Ferreira A.S., Nunes C., Castro A., Ferreira P., Coimbra M.A. (2014). Influence of grape pomace extract incorporation on chitosan films properties. Carbohyd. Polym..

[249] Genskowsky E., Puente L.A., Pérez-Álvarez J.A., Fernandez-Lopez J., Muñoz L.A., Viuda-Martos M. (2015). Assessment of antibacterial and antioxidant properties of chitosan edible films incorporated with maqui berry (Aristotelia chilensis). LWT-Food Sci. Technol..

[250] Sun L., Sun J., Chen L., Niu P., Yang X., Guo Y. (2017). Preparation and characterization of chitosan film incorporated with thinned young apple polyphenols as an active packaging material. Carbohyd. Polym..

[251] Serrano-León J.S., Bergamaschi K.B., Yoshida C.M., Saldaña E., Selani M.M., Rios-Mera J.D., Alencar S.M., Contreras-Castillo C.J. (2018). Chitosan active films containing agro-industrial residue extracts for shelf life extension of chicken restructured product. Food Res. Int..

[252] Kurek M., Garofulić I.E., Bakić M.T., Ščetar M., Uzelac V.D., Galić K. (2018). Development and evaluation of a novel antioxidant and pH indicator film based on chitosan and food waste sources of antioxidants. Food Hydrocoll..

[253] Talón E., Trifkovic K.T., Vargas M., Chiralt A., González-Martínez C. (2017). Release of polyphenols from starch-chitosan based films containing thyme extract. Carbohyd. Polym..

[254] Türk Baydır A., Aşçıoğlu Ç. (2018). Effects of Antioxidant Capacity and Peroxide Value on Oxidation Stability of Sunflower Oil. Chem. Mater..

[255] Berizi E., Hosseinzadeh S., Shekarforoush S.S., Barbieri G. (2018). Microbial, chemical, textural and sensory properties of coated rainbow trout by chitosan combined with pomegranate peel extract during frozen storage. Int. J. Biol. Macromol..

[256] Ahmed M., Pickova J., Ahmad T., Liaquat M., Farid A., Jahangir M. (2016). Oxidation of Lipids in Foods. Sarhad J. Agric..

[257] Khan S., Ranjha N.M. (2014). Effect of degree of cross-linking on swelling and on drug release of low viscous chitosan/poly (vinyl alcohol) hydrogels. Polym. Bull..

[258] Kandav G., Bhatt D., Jindal D.K. (2019). Formulation And Evaluation Of Allopurinol Loaded Chitosan Nanoparticles. Intern. J. Appl. Pharmaceutics.

[259] Ali A., Ahmed S. (2018). Recent advances in edible polymer based hydrogels as a sustainable alternative to conventional polymers. J. Agric. Food Chem..

[260] Gierszewska M., Ostrowska-Czubenko J., Chrzanowska E. (2018). Characteristics of ascorbic acid release from TPP-crosslinked chitosan/alginate polyelectrolyte complex membranes. Prog. Chem. Appl. Chitin Deriv..

[261] Valizadeh S., Naseri M., Babaei S., Hosseini S.M.H., Imani A. (2019). Development of bioactive composite films from chitosan and carboxymethyl cellulose using glutaraldehyde, cinnamon essential oil and oleic acid. Int. J. Biol. Macromol..

[262] Li C.G., Wang F., Peng W.G., He Y.H. (2013). Preparation of chitosan and epichlorohydrin cross-linked adsorbents and adsorption property of dyes. Proceedings of the Applied Mechanics and Materials 2013, Third International Conference on Applied Mechanics, Materials and Manufacturing (ICAMMM 2013).

[263] Kildeeva N.R., Kasatkina M.A., Mikhailov S.N. (2017). Peculiarities of obtaining biocompatible films based on chitosan cross linked by genipin. Polym. Sci. Ser. D.

[264] Zhang X., Do M.D., Casey P., Sulistio A., Qiao G.G., Lundin L., Lillford P., Kosaraju S. (2010). Chemical cross-linking gelatin with natural phenolic compounds as studied by high-resolution NMR spectroscopy. Biomacromolecules.

[265] Huber D., Tegl G., Baumann M., Sommer E., Gorji E.G., Borth N., Schleining G., Nyanhongo G.S., Guebitz G.M. (2017). Chitosan hydrogel formation using laccase activated phenolics as cross-linkers. Carbohyd. Polym..

[266] Silva-Weiss A., Bifani V., Ihl M., Sobral P.J.A., Gómez-Guillén M.C. (2013). Structural properties of films and rheology of film-forming solutions based on chitosan and chitosan-starch blend enriched with murta leaf extract. Food Hydrocoll..

[267] Lemes B.M., Novatski A., Ferrari P.C., Minozzo B.R., Justo A.D.S., Petry V.E.K., Vellosa J.C.R., Sabino S.d.R.F., Gunha J.V., Esmerino L.A. (2018). Physicochemical, biological and release studies of chitosan membranes incorporated with Euphorbia umbellata fraction. Rev. Bras. De Farmacogn..

[268] Aryal S., Baniya M.K., Danekhu K., Kunwar P., Gurung R., Koirala N. (2019). Total Phenolic Content, Flavonoid Content and Antioxidant Potential of Wild Vegetables from Western Nepal. Plants.

[269] Liu J., Liu S., Wu Q., Gu Y., Kan J., Jin C. (2017). Effect of protocatechuic acid incorporation on the physical, mechanical, structural and antioxidant properties of chitosan film. Food Hydrocoll..

[270] Smith R.A., Walker R.C., Levit S.L., Tang C. (2019). Single-Step Self-Assembly and Physical Crosslinking of PEGylated Chitosan Nanoparticles by Tannic Acid. Polymers.

[271] Rivero S., García M.A., Pinotti A. (2010). Crosslinking capacity of tannic acid in plasticized chitosan films. Carbohyd. Polym..

[272] Azeredo H.M., Waldron K.W. (2016). Crosslinking in polysaccharide and protein films and coatings for food contact—A review. Trends Food Sci. Technol..

[273] Qin Y.Y., Zhang Z.H., Li L., Yuan M.L., Fan J., Zhao T.R. (2015). Physio-mechanical properties of an active chitosan film incorporated with montmorillonite and natural antioxidants extracted from pomegranate rind. J. Food Sci. Technol..

[274] Mayachiew P., Devahastin S. (2010). Effects of drying methods and conditions on release characteristics of edible chitosan films enriched with Indian gooseberry extract. Food Chem..

[275] Hauser C., Peñaloza A., Guarda A., Galotto M.J., Bruna J.E., Rodríguez F.J. (2016). Development of an Active Packaging Film Based on a Methylcellulose Coating Containing Murta (Ugni molinae Turcz) Leaf Extract. Food Bioprocess. Technol..

[276] Liu B., Xu H., Zhao H., Liu W., Zhao L., Li Y. (2017). Preparation and characterization of intelligent starch/PVA films for simultaneous colorimetric indication and antimicrobial activity for food packaging applications. Carbohyd. Polym..

[277] Ghelejlu S.B., Esmaiili M., Almasi H. (2016). Characterization of chitosan–nanoclay bionanocomposite active films containing milk thistle extract. Int. J. Biol. Macromol..

